# Transcriptomic and metabolomic analysis reveals the role of CoA in the salt tolerance of *Zygophyllum* spp

**DOI:** 10.1186/s12870-019-2226-8

**Published:** 2020-01-06

**Authors:** Jie Wang, Xi Jiang, Chufeng Zhao, Zhongming Fang, Peipei Jiao

**Affiliations:** 1 Key Laboratory of Biological Resource Protection and Utilization of Tarim Basin, Xinjiang Production and Construction Group, Alar, 843300 China; 2 College of Life Sciences, Tarim University, Alar, 843300 China; 3 Center of Applied Biotechnology, Wuhan University of Bioengineering, Wuhan, 430415 China; 4 College of Plant Sciences, Tarim University, Alar, 843300 China; 5 State Key Laboratory of Hybrid Rice, Engineering Research Center for Plant Biotechnology and Germplasm Utilization of Ministry of Education, College of Life Sciences, Wuhan University, Wuhan, 430072 China; 6 College of Agricultural Sciences, Guizhou University, Guiyang, 550025 China

**Keywords:** *Zygophyllum*, Salt stress, Transcriptomics, Metabolomics, Branched-chain-amino-acid aminotransferase, CoA

## Abstract

**Background:**

*Zygophyllum* is an important medicinal plant, with notable properties such as resistance to salt, alkali, and drought, as well as tolerance of poor soils and shifting sand. However, the response mechanism of *Zygophyllum* spp. to abiotic stess were rarely studied.

**Results:**

Here, we aimed to explore the salt-tolerance genes of *Zygophyllum* plants by transcriptomic and metabolic approaches. We chose *Z. brachypterum*, *Z. obliquum* and *Z. fabago* to screen for salt tolerant and sensitive species. Cytological observation showed that both the stem and leaf of *Z. brachypterum* were significantly thicker than those of *Z. fabago.* Then, we treated these three species with different concentrations of NaCl, and found that *Z. brachypterum* exhibited the highest salt tolerance (ST), while *Z. fabago* was the most sensitive to salt (SS). With the increase of salt concentration, the CAT, SOD and POD activity, as well as proline and chlorophyll content in SS decreased significantly more than in ST. After salt treatment, the proportion of open stomata in ST decreased significantly more than in SS, although there was no significant difference in stomatal number between the two species. Transcriptomic analysis identified a total of 11 overlapping differentially expressed genes (DEGs) in the leaves and roots of the ST and SS species after salt stress. Two branched-chain-amino-acid aminotransferase (BCAT) genes among the 11 DEGs, which were significantly enriched in pantothenate and CoA biosynthesis, as well as the valine, leucine and isoleucine biosynthesis pathways, were confirmed to be significantly induced by salt stress through qRT-PCR. Furthermore, overlapping differentially abundant metabolites showed that the pantothenate and CoA biosynthesis pathways were significantly enriched after salt stress, which was consistent with the KEGG pathways enriched according to transcriptomics.

**Conclusions:**

In our study, transcriptomic and metabolomic analysis revealed that BCAT genes may affect the pantothenate and CoA biosynthesis pathway to regulate the salt tolerance of *Zygophyllum* species, which may constitute a newly identified signaling pathway through which plants respond to salt stress.

## Background

Soil salinization has become an important global ecological and environmental problem, and the salinized soil affects the geographical distribution of plants, restricts their productivity, and threatens food security [1]. High salt levels can cause secondary stresses such as ion toxicity, hyperosmolar stress and oxidative damage, which seriously affect the growth and development of plants [2]. To adapt to the salinized environment, plants initiate a series of regulatory mechanisms to reduce salt damage, including morphological changes [3, 4], osmotic regulation in cells [5, 6], scavenging of reactive oxygen species [7], excretion and intracellular compartmentalization of salts [8, 9], regulation of potassium transport [10, 11], regulation of aquaporin expression [12, 13], and changes in the rate of photosynthesis [14].

To understand the molecular mechanisms of plant salt tolerance, many researchers focused on screening salt-tolerant species to obtain salt-tolerance genes. Recently, increasing numbers of studies have elucidated the salt stress signal transduction pathways of plants, which are important for the comprehensive understanding of the molecular mechanisms of plant salt tolerance. Abscisic acid (ABA) plays a key role in optimizing plant water use, and it is essential for seed development and responses to drought, high salinity and other environmental stresses [15]. ABA is widely believed to be involved in osmotic stress signal transduction under salt stress [16]. Protein phosphorylation is a central feature of eukaryotic signal transduction, and it also plays a role in osmotic stress adaptation [2]. The mitogen-activated protein kinase (MAPK) pathway not only participates in plant growth and development processes, but can also be activated by a number of different biological and abiotic stresses, such as high salt, drought, and cold stress [17, 18]. The salt overly sensitive (SOS) signaling pathway is responsible for Na^+^ excretion in plant root cells, which is one of the best-studied mechanisms of plant salt tolerance. In this pathway, three SOS genes (SOS1, SOS2 and SOS3) are involved in mediating the signals that regulate the intracellular ion balance [19–22].

*Zygophyllum* is a genus whose members are mainly distributed in desert and semi-desert areas, and consequently have a strong ability to adapt to salt and drought stress. Therefore, *Zygophyllum* plants have important genetic resources that protect them from abiotic stresses, especially salt stress. Furthermore, *Zygophyllum* plants are considered to be early colonizers of soils polluted with heavy metals under semiarid conditions, and they have broad-spectrum resistance to heavy metals such as Pb, Zn and Cu [23, 24]. To date, there were only a few studies on the basic biology of *Zygophyllum*, and systematic investigations of its unique salt-tolerance characteristics at the seedling stage are extremely limited. In this study, the salt tolerance of three *Zygophyllum* species (*Z*. *brachypterum*, *Z*. *obliquum* and *Z. fabago*) was assessed to screen the salt-tolerant and salt-sensitive species. Then, transcriptomic and metabolomic analyses were performed to identify the genes and metabolic pathways involved in salt tolerance. The results showed that two branched-chain-amino-acid aminotransferase (BCAT) genes, which were the most likely candidate genes to respond to salt stress, might affect the CoA biosynthesis pathway to regulate the salt tolerance of *Zygophyllum* species.

## Results

### Anatomical differences of three *Zygophyllum* species

**Fig. 1:**
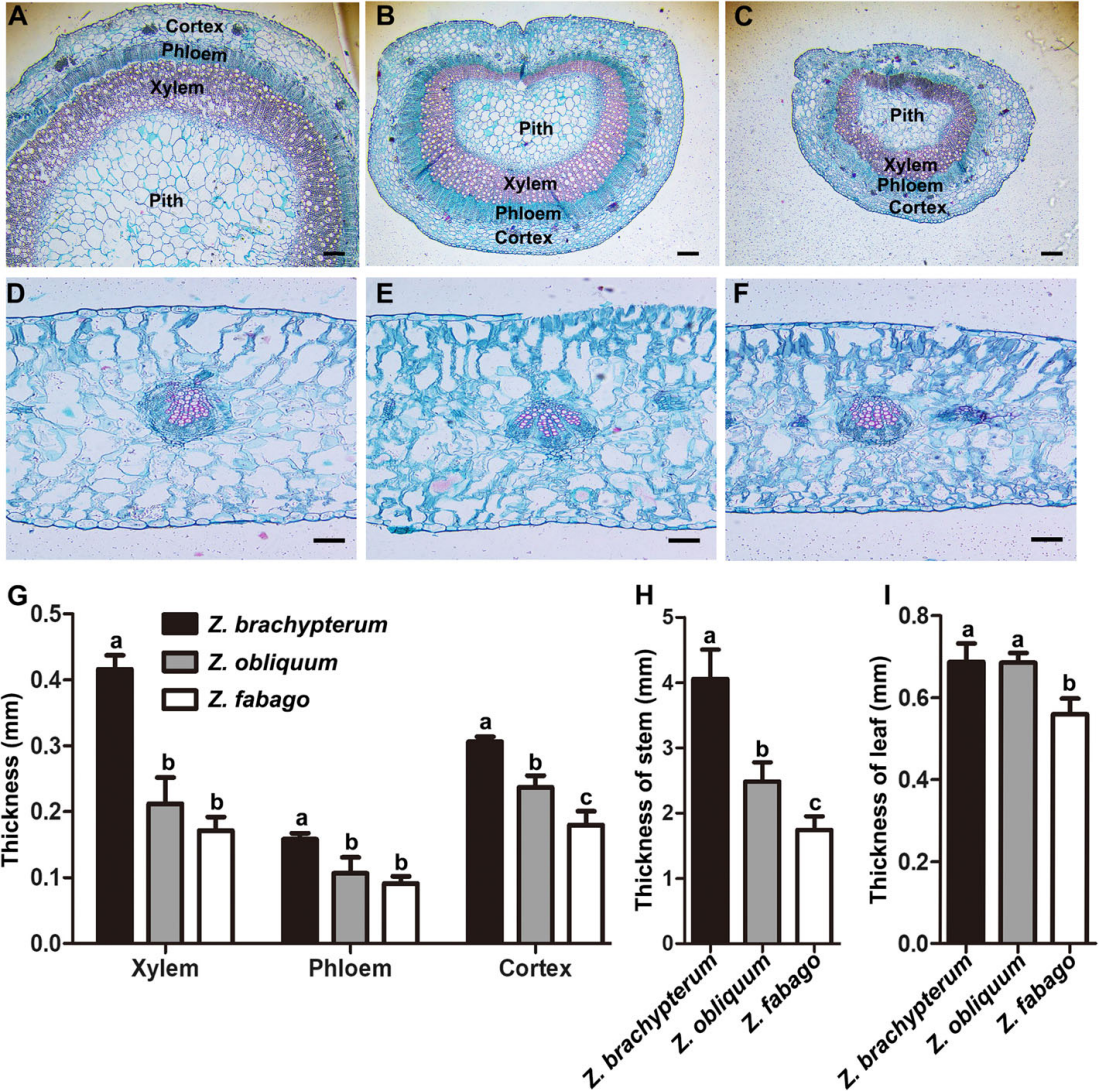
Histological analysis of three *Zygophyllum* species. Cross section analysis of the main stem of *Zygophyllum brachypterum* (**a**), *Zygophyllum obliquum* (**b**) and *Zygophyllum fabago* (**c**). Cross section analysis of the leaf of *Zygophyllum brachypterum* (**d**), *Zygophyllum obliquum* (**e**) and *Zygophyllum fabago* (**f**). Statistical analysis of the thickness of main stem xylem, phloem and cortex (**g**). Statistical analysis of the thickness of the main stem (**h**) and leaf (**i**). Scale bars = 0.2 mm in (**a**), (**b**) and (**c**). Scale bars = 0.1 mm in (**d**), (**e**) and (**f**). Values are the means ± SD from three replicates. Different letters indicate significant differences at *P* < 0.05 according to Duncan’s multiple range test

When plants adapt to a saline soil, their morphology also changes. We analyzed the anatomical structure of main stems and leaves of *Z. brachypterum*, *Z. obliquum* and *Z. fabago* under the conditions of the natural environment. The results demonstrated that the pith area of the main stem of *Z. brachypterum* was the largest of the three species, while that of *Z. fabago* was the smallest (Fig. 1a-c). The leaves of *Z. fabago* were the thinnest among the three species, while no significant difference was found between *Z. brachypterum* and *Z. obliquum* (Fig. 1d-f). Statistical analysis showed that the thicknesses of the xylem, phloem and cortex of *Z. brachypterum* were all significantly larger than those of *Z. fabago* (Fig. 1g), in accordance with the thickest stem of *Z. brachypterum* and the thinnest stem of *Z. fabago* (Fig. 1f). In addition, the leaves of *Z. brachypterum* and *Z. obliquum* were significantly thicker than those of *Z. fabago* (Fig. 1i), which indicated that the leaves of *Z. brachypterum* and *Z. obliquum* might have a higher water storage capacity. These results indicated that *Z. brachypterum* plants might have a higher capacity to transport water from the stems and store it in the leaves than *Z. fabago*.

### Salt resistance of the three *Zygophyllum* species

**Fig. 2:**
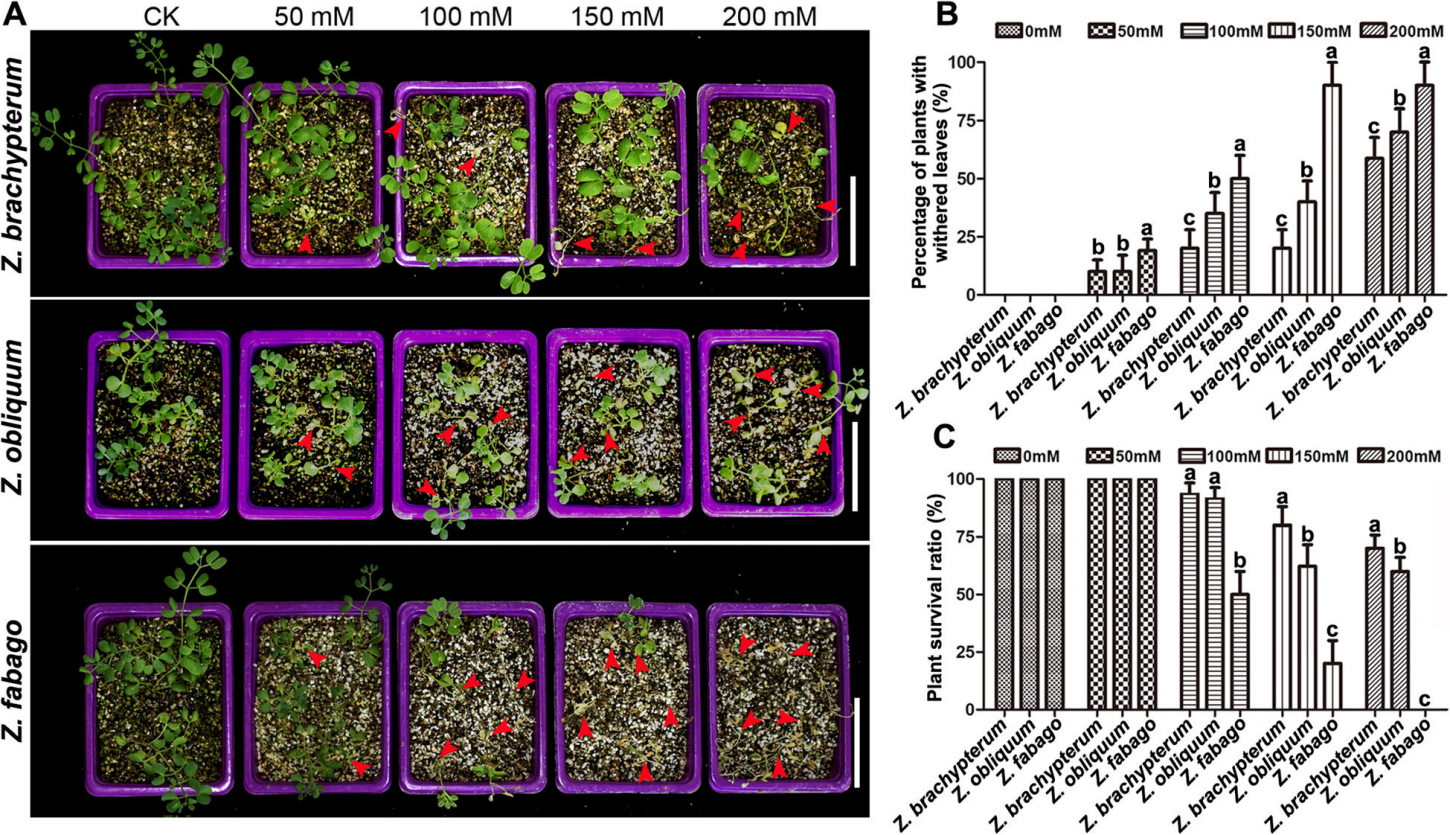
Analysis of the salt tolerance of three *Zygophyllum* species. **a,** Phenotypic analysis of the three *Zygophyllum* species treated with 0 mM NaCl (CK, control), 50 mM NaCl, 100 mM NaCl, 150 mM NaCl and 200 mM NaCl. Scale bars = 10 cm. **b**, Percentage of plants with withered leaves. **c**, Statistical analysis of the plant survival rate of the three *Zygophyllum* species. The values are the means ± SD from three replicates. Different letters indicate significant differences at *P* < 0.05 according to Duncan’s multiple range test

As desert plants, *Zygophyllum* spp. have good resistance against abiotic stresses. To discover the differences in salt stress tolerance among the three *Zygophyllum* species, we treated seedlings of *Z. brachypterum*, *Z. obliquum* and *Z. fabago* with 50 NaCl, 100 NaCl, 150 NaCl and 200 mM NaCl, or left them untreated (control, CK). Compared with the control group, the leaves of the three *Zygophyllum* species all showed different degrees of wilting as the salt concentration increased, whereby the degree of leaf wilting was the highest in *Z. fabago* and lowest in *Z. brachypterum* (Fig. 2a and b). Moreover, seedling growth of *Z. obliquum* and *Z. fabago* was significantly inhibited by 50 mM NaCl (Fig. 2a). With the further increase of salt concentration, the seedlings began to die, and the plant survival rate was dramatically decreased in *Z. fabago* (Fig. 2a and c). Conversely, the seedling survival rate of *Z. brachypterum* was the highest, even when the NaCl concentration was increased above 150 mM (Fig. 2c). These results showed that *Z. brachypterum* had the strongest tolerance to salt stress, while *Z. fabago* had the weakest salt tolerance.

**Fig. 3:**
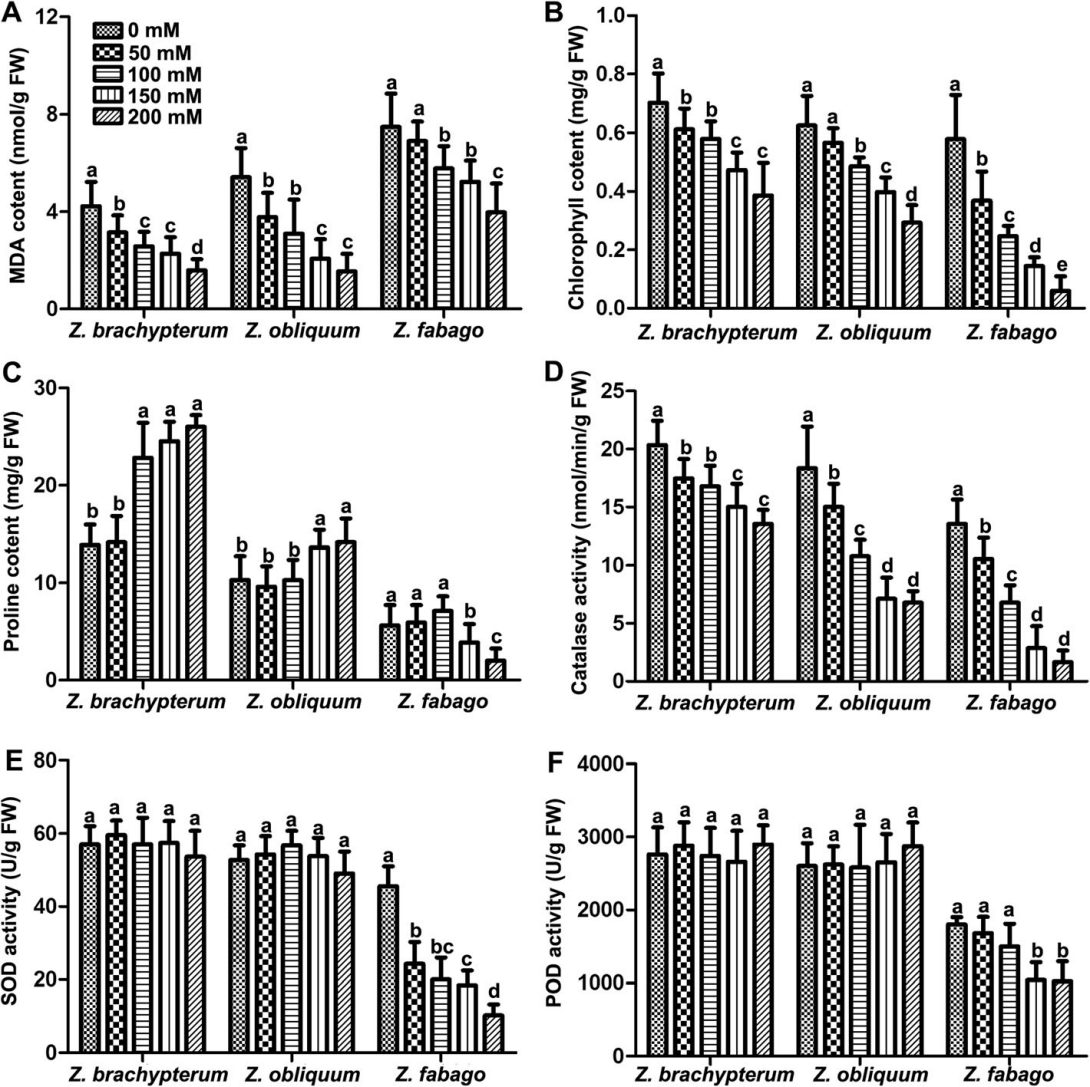
Determination of physiological indices of the three *Zygophyllum* species under salt stress. MDA content (**a**), chlorophyll content (**b**), proline content (**c**), CAT activity (**d**), SOD activity (**e**) and POD (**f**) activity in leaves of three *Zygophyllum* species treated with different concentrations of NaCl. The values are the means ± SD from three replicates. Different letters indicate significant differences at *P* < 0.05 according to Duncan’s multiple range test

Under salt stress, a number of physiological indices are usually affected in plants, such as the proline, malondialdehyde (MDA) and chlorophyll content, as well as superoxide dismutase (SOD), catalase (CAT) and peroxidase (POD) activity. Two weeks after the final treatment with NaCl, we measured these physiological indices in the three *Zygophyllum* species at different salt concentrations. The results showed that the MDA content decreased dramatically with the increase of salt concentration, especially in *Z. brachypterum* and *Z. obliquum*, but more slowly in *Z. fabago* (Fig. 3a). However, the chlorophyll content decreased dramatically in *Z. fabago* and the decrease was slowest in *Z. brachypterum* (Fig. 3b). The proline content increased significantly in Z. *brachypterum* when the salt concentration was higher than 100 mM, while in *Z. obliquum* it increased significantly when the salt concentration was higher than 150 mM. The degree of increase was not as great as that of *Z. brachypterum*. Interestingly, the proline content in *Z. fabago* decreased significantly when the salt concentration was higher than 150 mM (Fig. 3c). CAT activity decreased with the increase of salt concentration in all the *Zygophyllum* species, whereby the reduction was the largest in *Z. fabago* and smallest in *Z. brachypterum* (Fig. 3d). The reduction of SOD and POD activity was also largest in *Z. fabago*, while no significant difference was found in either *Z. brachypterum* or *Z. obliquum* among the groups treated with different NaCl concentrations (Fig. 3e and f). These results indicated that *Z. brachypterum* was the most salt-tolerant (ST) species, while *Z. fabago* was the most salt-sensitive (SS).

### Salt induced stomatal closure in the ST species

**Fig. 4:**
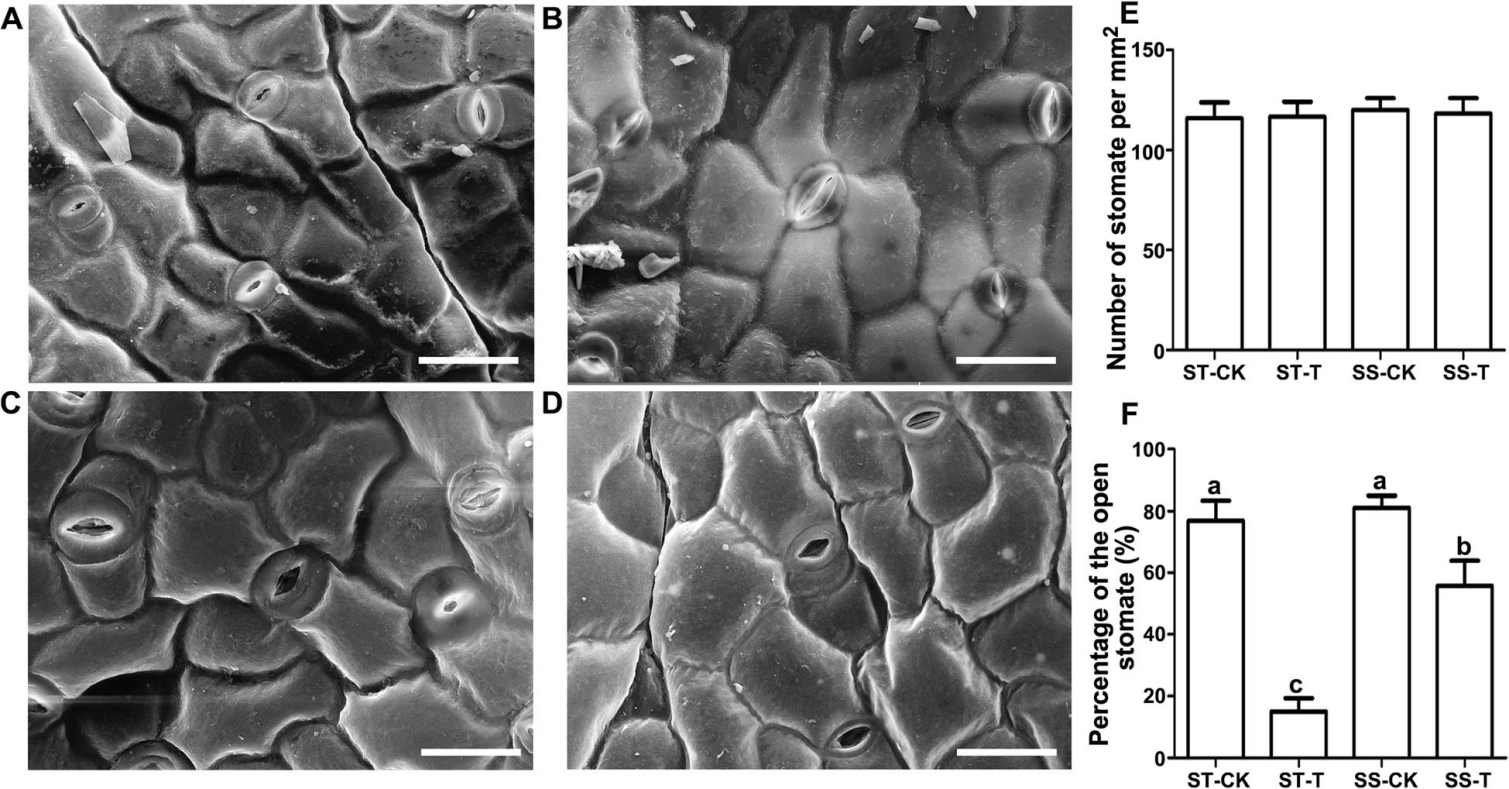
SEM analysis of the differences of the leaf epidermis between the ST and SS *Zygophyllum* species. Seedlings at the 10-leaf stage were treated with 150 mM NaCl for 24 h, after which their leaf epidermis was observed by SEM. Leaf epidermis of the ST species after treatment with 0 mM (**a**) and 150 mM NaCl (**b**). Leaf epidermis of the SS species after treatment with 0 mM (**c**) and 150 mM NaCl (**d**). Statistical analysis of the number of stomata (**e**) and the percentage of open stomata (**f**). Scale bars = 50 μm in (**a**-**d**). The values are the means ± SD from three replicates. Different letters indicate significant differences at *P* < 0.05 according to Duncan’s multiple range test. The absence of marked significance in **e** indicates that there were no significant differences between any of the groups

Stomatal movement regulates photosynthesis and transpiration, and plays an important role in regulating plant growth and development in response to abiotic stresses such as salt and drought. To understand the difference of stomatal movement after salt treatment between the ST and SS species, we treated ST and SS seedlings (10-leaf stage) with 150 mM NaCl for 24 h, after which leaves from the same position on the main stem were selected to perform scanning electron microscopy (SEM). The results demonstrated that there was no significant difference in the number of stomata per square millimeter between the salt treatment group and the control group of either species (Fig. 4a-e). We also measured the percentage of open stomata in the control and salt-treated groups of the ST and SS species. The results showed that open stomata accounted for 80% of the total in the control group but only 20% in the salt-treated group of the ST species. By contrast, open stomata still accounted for 80% of the control group but 60% in the salt-treated group of the SS species (Fig. 4f). These results indicated that a sharp decrease in the ratio of open stomata was conducive to the retention of water in the leaves of the ST species under salt stress.

### Analysis of differentially expressed genes (DEGs) between the ST and SS species

In order to reveal the mechanism underlying the difference of salt tolerance between the ST and SS species, 24 RNA-seq libraries containing three biological replicates, stratified by the absence or presence of 150 mM NaCl treatment, were constructed from roots and leaves. The high-throughput RNA-seq generated 43.8 to 65.2 million raw reads for each sample (Additional file 1: Table S1). After removing reads containing adapters, reads containing ploy-N and low-quality reads from the raw data, the number of clean reads was higher than 42.7 million for each sample. About 62.63–83.73% of the clean reads from each sample were mapped to the *Arabidopsis* reference genome using RSEM software (Additional file 1: Table S1). Using the Trinity platform, 498,605 unigenes were reassembled from the clean reads. The length of all unigenes varied from 201 to 37,056 bp, with an average length of 1038 bp (Additional file 1: Table S2). The size distribution of the unigenes is illustrated in Additional file 1: Figure S1. The constructed unigene dataset was used as reference for further analysis, and was deposited with the national center for biotechnology information (NCBI).

**Fig. 5:**
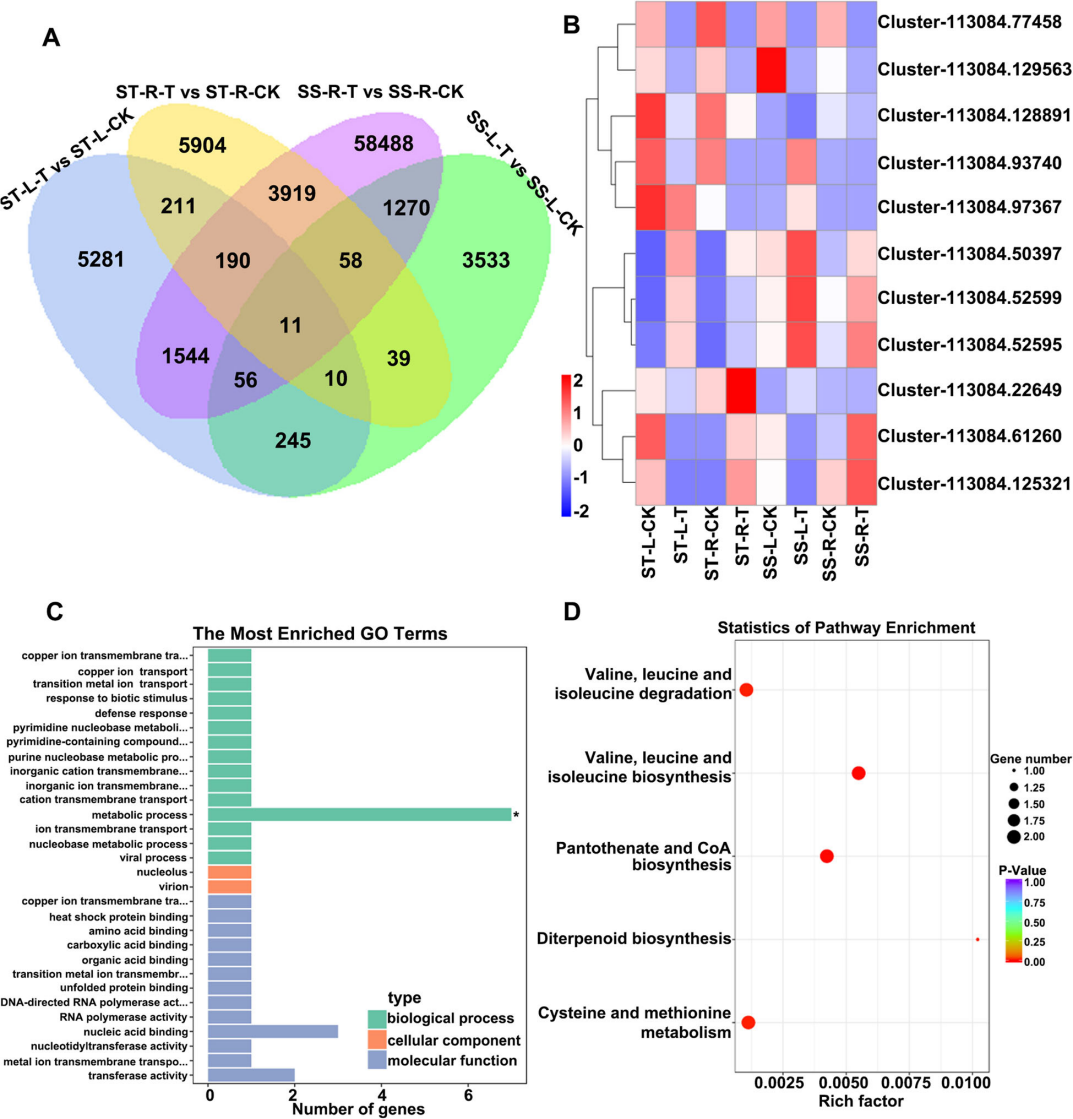
Analysis of DEGs between the ST and SS species in the leaves and roots. **a**, Overlap of DEGs under control and salt stress conditions. L, leaf; R, root; T, treatment with 150 mM NaCl; CK, control (no added NaCl). **b**, Expression patterns of the 11 overlapping DEGs between the ST and SS species. The heatmap presents normalized FPKM expression values. **c**, Analysis of GO terms based at the 11 overlapping DEGs. **d**, KEGG pathway analysis based at the 11 overlapping DEGs

DEGs between the control and NaCl-treated groups were analyzed using the R package DESeq2 based on the criteria *P* < 0.05 and |log2FoldChange| > 1. Compared to the control group, there were 8088 and 5272 DEGs in the leaves, as well as 10,392 and 66,743 DEGs in the roots of the salt-treated groups of the ST and SS species (Additional file 1: Figure S2). A total of 322 overlapping DEGs in the leaves and 4178 in the roots were found both in the ST and SS plants after salt treatment, compared to each control group (Fig. 5a). The expression of many DEGs was decreased or induced after salt treatment, both in leaves (Additional file 1: Figure S3A) and roots (Additional file 1: Figure S4A). GO term analysis showed that the DEGs in the leaves were mostly enriched in molecular functions, including sequence-specific DNA binding, nucleic acid binding transcript, and transcription factor activity (Additional file 1: Figure S3B), while those from roots were mostly enriched in cellular component categories such as cell, cell part and intracellular (Additional file 1: Figure S4B). KEGG pathway enrichment analysis indicated that the DEGs in the leaves significantly affected plant hormone signal transduction, nitrogen metabolism, as well as glyoxylate and dicarboxylate metabolism (Additional file 1: Figure S3C), while only plant hormone signal transduction was significantly affected in the roots (Additional file 1: Figure S4C). Additionally, expression of some DEGs was validated by qRT-PCR both in leaves (Additional file 1: Figures S5A and B) and roots (Additional file 1: Figure S5C and D), and the results showed that the expression level of over 90% genes was consistent with the FPKM values of RNA-seq. These results indicated that the DEGs might affect phytohormones levels in the roots and leaves, which in turn affected the salt tolerance of *Zygophyllum* plants.

### Candidate salt tolerance genes of *Zygophyllum*

**Fig. 6:**
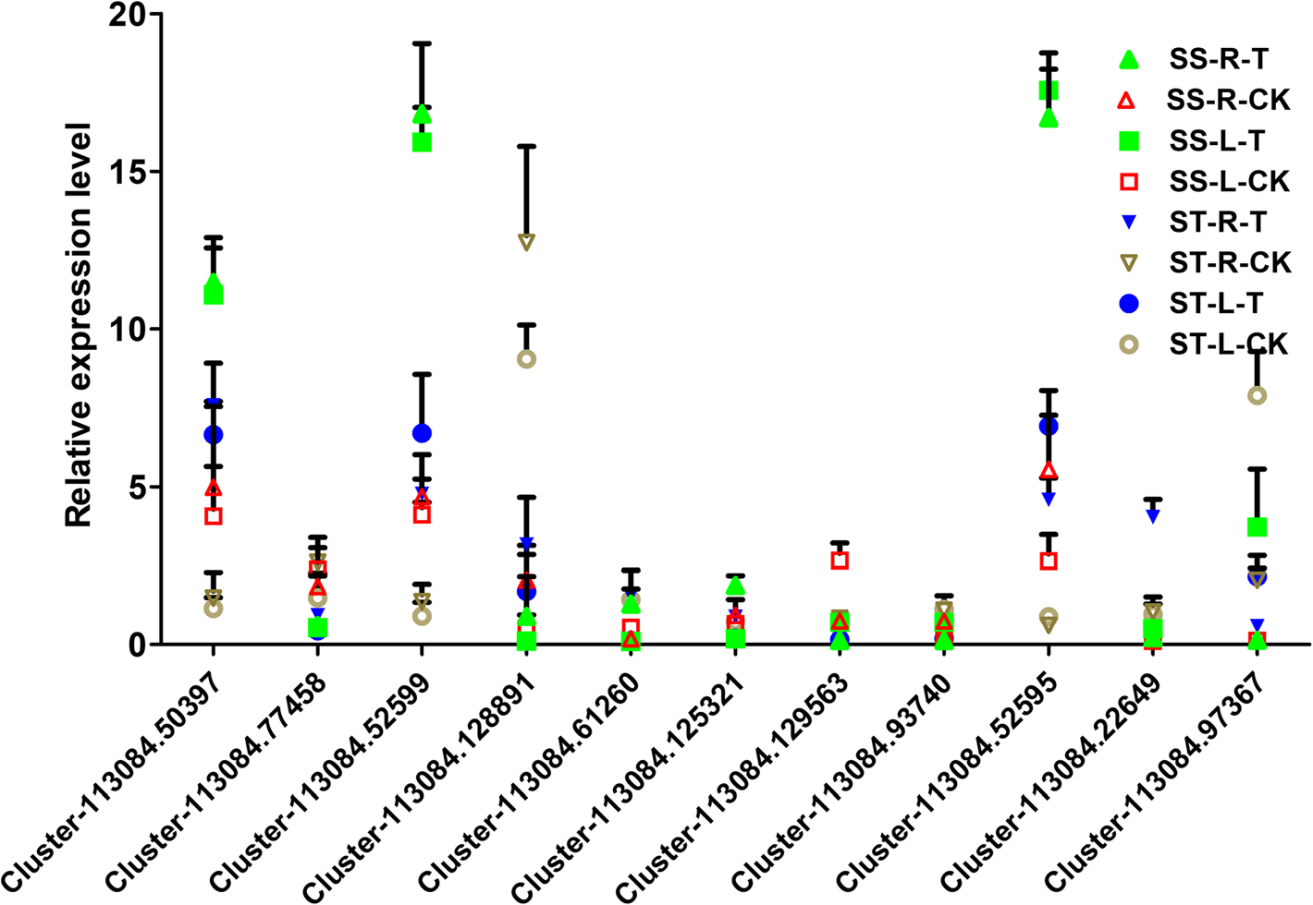
Validation of the expression of the 11 overlapping DEGs by qRT-PCR. Different symbols represented different groups of the ST and SS species under control and salt stress conditions. L, leaf; R, root; T, treatment with 150 mM NaCl; CK, control (no added NaCl). The values are the means ± SD from three replicates

To further screen candidate genes responsible for salt tolerance, we combined the DEGs between the treatment and control groups in the roots and leaves. There were only 11 overlapping DEGs in roots and leaves, which exhibited different expression patterns (Fig. 5a and b). Among them, the expression of two genes (Cluster-113,084.52599 and Cluster-113,084.52595) was higher in both leaves and roots of the salt treatment groups than in the corresponding control groups (Fig. 5b), which indicated that these two genes were induced by salt, especially in the SS species. The gene function annotation indicated that both Cluster-113,084.52599 and Cluster-113,084.52595 encoded branched-chain amino acid aminotransferases (BCAT, Table 1). GO term analysis revealed that the 11 overlapping DEGs were mostly enriched in biological processes, especially in metabolism (Fig. 5c). KEGG pathway analysis demonstrated that 2 BCAT genes of the 11 overlapping DEGs were significantly enriched in valine, leucine and isoleucine biosynthesis, as well as the pantothenate and CoA biosynthesis pathways (Table 1 and Fig. 5c). To validate the expression levels of the 11 overlapping DEGs, qRT-PCR was performed. The results revealed that the expression trends of these genes in different groups were consistent with the FPKM values of RNA-seq. Moreover, the two BCAT genes were strongly induced by salt stress, and the highest expression was observed in the SS species (Fig. 6). Taken together, the results indicate that the two BCAT genes might be candidate genes that play key roles in the regulation of salt tolerance in *Zygophyllum* by affecting the biosynthesis of valine, leucine and isoleucine, as well as pantothenate and CoA.

**Table 1:** Functional annotation of the 11 overlapping genes between th ST and SS species. NA indicates not available

Gene ID	Function Description	KEGG pathway
Cluster-113,084.50397	Outer envelope pore protein 16–2	NA
Cluster-113,084.77458	ACT domain-containing protein ACR8	NA
Cluster-113,084.52599	Branched-chain-amino-acid aminotransferase 2	Valine, leucine and isoleucine metabolismPantothenate and CoA biosynthesisCysteine and methionine metabolism
Cluster-113,084.128891	Gibberellin 20 oxidase 2	Diterpenoid biosynthesis
Cluster-113,084.61260	Probable LRR receptor-like serine/threonine-protein kinase	NA
Cluster-113,084.125321	Protein argonaute 4	NA
Cluster-113,084.129563	electron transporter, putative	NA
Cluster-113,084.93940	Chaperone protein	NA
Cluster-113,084.52595	Branched-chain-amino-acid aminotransferase 2	Valine, leucine and isoleucine metabolismPantothenate and CoA biosynthesisCysteine and methionine metabolism
Cluster-113,084.22649	Short-chain dehydrogenase TIC 32	NA
Cluster-113,084.97367	MLP-like protein 34	NA

### Metabolomic analysis of differences between the ST and SS species

**Fig. 7:**
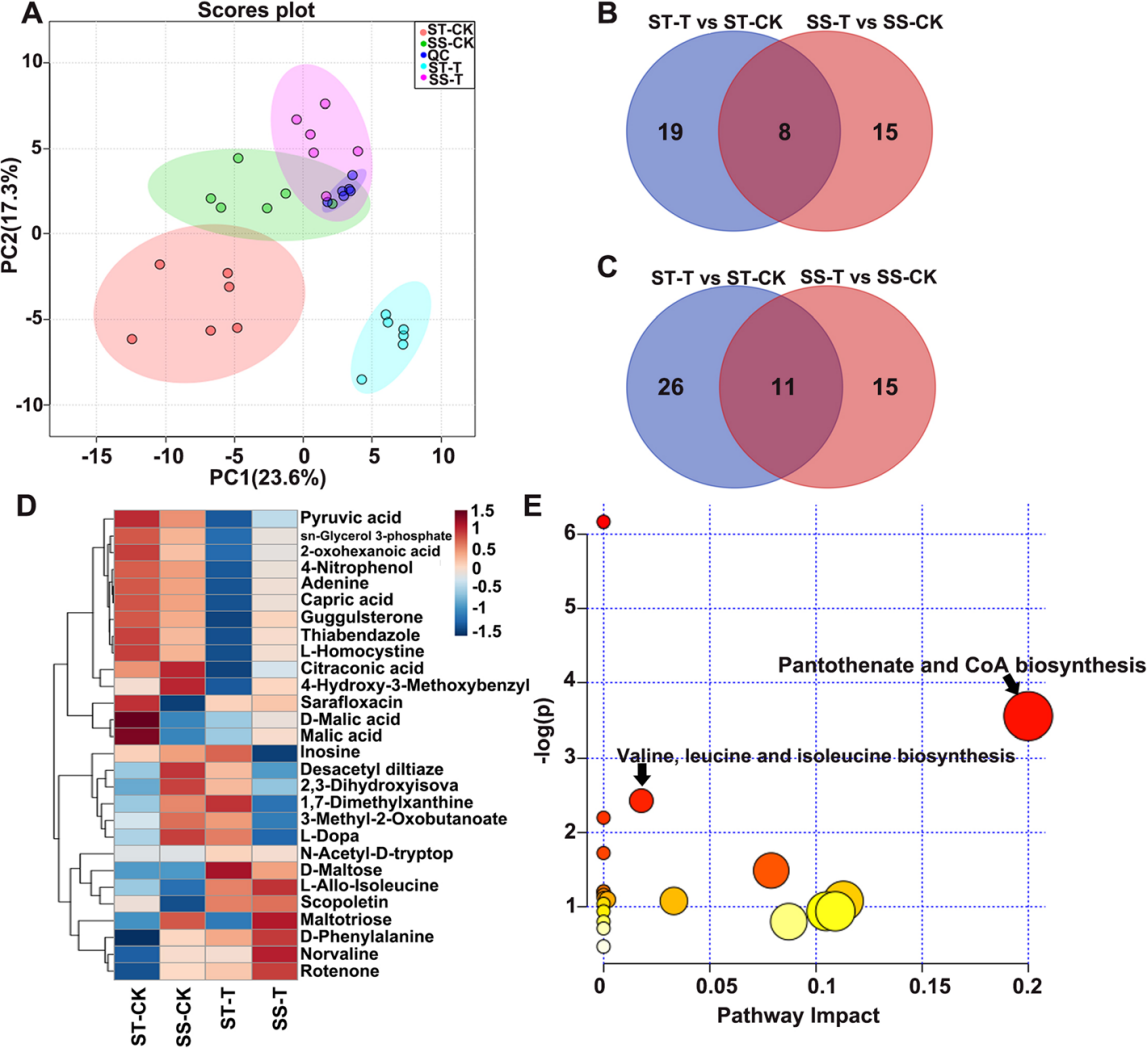
Metabolomic analysis of differences between the SS and ST species. **a**, Principal component analysis (PCA) of the metabolic profiles of the ST and SS species under control and salt stress conditions. The explained variance proportions are shown in brackets. QC, quality control. **b**, Overlap among upregulated metabolites in the ST and SS species under control and salt stress conditions. **c**, Overlap among the downregulated metabolites in the ST and SS species under the control and salt stress conditions. **d**, Heatmap illustrating the Log^2^(fold change) of selected overlapping differentially abundant metabolites using Euclidean distance measure and average clustering. **e**, KEGG pathway analysis based at the overlapped different abundant metabolites. T, treatment with 150 mM NaCl; CK, control (no added NaCl)

As GO term analysis of the 11 common DEGs between ST and SS showed that metabolic processes were significantly enriched, we compared the metabolic profiles of the leaves of the SS and ST species under control and 150 mM NaCl stress conditions. In order to ensure the reliability of the experimental data and results, we conducted 6 repetitions for each group. A total of 315 metabolites could be identified in all samples from the ST and SS species. According to the principal component analysis (PCA), there was an obvious separation between samples within treatments and the controls of the SS and ST species (Fig. 7a). Compared to the control group, a total of 70 and 55 differentially abundant metabolites were identified in leaves of the ST and SS salt-treated groups, among which 8 overlapping upregulated and 11 overlapping downregulated metabolites between the ST and SS salt-treated groups were identified (Fig. 7b and c). Additionally, another 9 differentially abundant metabolites exhibited the opposite regulation pattern between the ST and SS species (Fig. 7d). Subsequent KEGG analysis based on the 28 overlapping differentially abundant metabolites showed that the pantothenate and CoA biosynthesis pathway was significantly enriched (Fig. 7e). Under salt stress, an important intermediate of CoA synthesis, 3-methyl-2-oxobutanoate, was significantly upregulated in the ST, but downregulated in the SS species (Additional file 1: Figure S6A). Additionally, valine, which can be produced from 3-methyl-2-oxobutanoate under the action of BCATs, was significantly upregulated in the SS species under salt stress (Additional file 1: Figure S6B). There was no significant difference in the CoA content between the ST salt-treated group and the corresponding control group, while the CoA content of the SS salt-treated group was significantly downregulated (Additional file 1: Figure S6C), which indicated that the CoA content was very important for *Zygophyllum* salt tolerance.

**Fig. 8:**
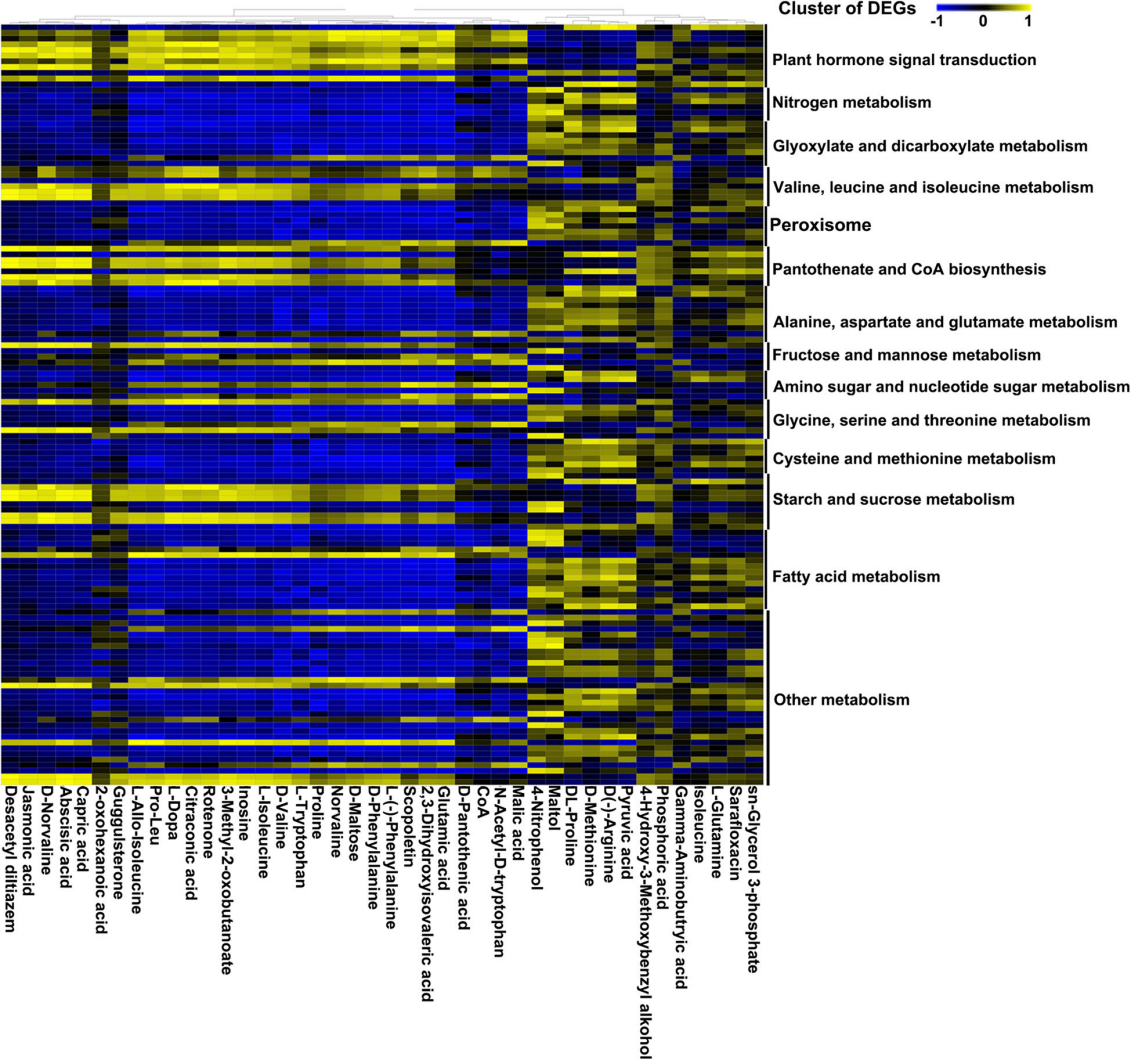
Correlation analysis between DEGs and leaf physiological characteristics of ST specie under control and salt stress conditions. A total of 134 DEGs and 42 metabolites were used for correlation matrix analysis. The coordinated shift in metabolites was evaluated by pair correlation analysis. The correlation coefficient (positive or negative) is represented by virtual color as indicated in the color key

To identify DEGs that were functionally related with leaf physiological characteristics, two groups of correlation analysis (ST salt-treated vs. ST control and SS salt-treated vs. SS control) between 134 DEGs and 42 metabolites were performed in this study. The coordinated shift in metabolites of ST species under control and salt stress conditions showed that most of pantothenate and CoA biosynthesis signaling-related genes are positively correlated with 3-methyl-2-oxobutanoate, and 3-methyl-2-oxobutanoate was positively correlated with most of genes related to plant hormone signal transduction, valine, leucine and isoleucine metabolism, and starch and sucrose metabolism (Fig. 8). The results of coordinated shift in metabolites of SS species under control and salt stress conditions were opposite to those of ST species (Additional file 1: Figure S7). It is suggested that salt treatment could induce up-regulation of some genes related to a series of metabolic pathways in the ST species, which resulted in a significant increase in the content of 3-methyl-2-oxobutanoate to maintain a relatively high content of CoA in ST species. Therefore, the pantothenate and CoA biosynthesis pathways might play an important role in the regulation of salt tolerance in *Zygophyllum* plants.

## Discussion

*Zygophyllum* is a genus of mostly perennial and rarely annual herbs in its own family, *Zygophyllaceae*. There are about 100 species of this genus in the world, mainly distributed in deserts, grasslands and the desertification-affected grassland belt of central Asia, the Mediterranean coast, Africa and Australia [25]. *Zygophyllum* plants were reported to have certain medicinal value, such as antihypertensive [26], hypoglycemic [27, 28], anti-inflammatory and antibacterial activity [29, 30]. In addition to their medicinal value, *Zygophyllum* plants also have excellent resistance to stress. Due to the long-term adaptation to the natural arid environment, *Zygophyllum* possesses excellent resistance to drought, salt, alkali and heavy metals, as well as being tolerant to poor soil, wind erosion, and shifting sands.

In this study, we screened the salt-tolerant species *Z. brachypterum* and the salt-sensitive species *Z. fabago* among three *Zygophyllum* species. In order to uncover the salt-tolerance mechanism, transcriptomic and metabolic comparisons between *Z. brachypterum* and *Z. fabago* were performed. Interestingly, DEGs in the roots and leaves of the salt-treated groups compared with the respective control groups were significantly enriched in the hormone signal transduction pathways, which indicated that phytohormones might also play an important role in the regulation of *Zygophyllum* salt tolerance. However, only 11 overlapping DEGs were identified when the DEGs in roots and leaves were combined. Among these, 2 BCATs were significantly enriched in valine, leucine and isoleucine biosynthesis, as well as the pantothenate and CoA biosynthesis pathways. These results indicated that the 11 overlapped DEGs in roots and leaves might affect *Zygophyllum* salt tolerance by affecting valine, leucine and isoleucine biosynthesis, as well as the pantothenate and CoA biosynthesis pathways.

**Fig. 9:**
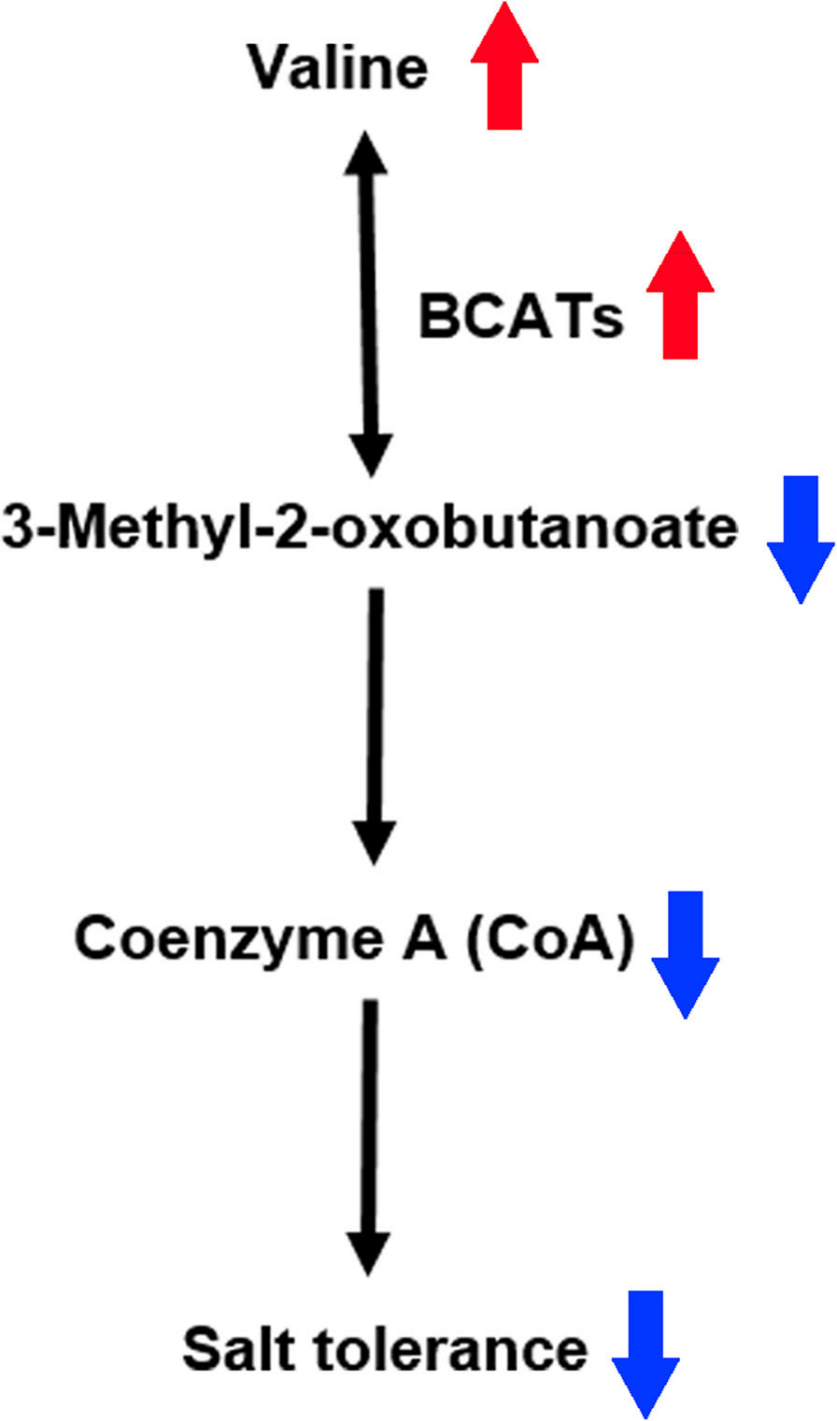
A proposed model for the salt tolerance mechanism of *Zygophyllum* plants. The red upward arrows indicate upregulation, and the blue downward arrows indicate downregulation

It has been known for a long time that BCATs catalyse the last step of the synthesis and/or the initial step of the degradation of leucine, isoleucine and valine [31]. The intermediates of branched-chain amino acid biosynthesis are also substrates of pantothenate and CoA synthesis [32]. Furthermore, degradation of branched-chain amino acids by BCATs under drought stress conditions may help maintain the pool of free branched-chain amino acids at low and non-toxic levels [33], which indicated that BCAT genes play an important role in tolerance to abiotic stresses. According to the analysis of the overlapping differentially abundant metabolites between the ST and SS species, the pantothenic acid and CoA biosynthesis pathway was significantly enriched. CoA is an important cofactor in many biosynthesis, degradation and energy generation pathways [34]. Additionally, the CoA biosynthetic enzyme phosphopantetheine adenylyltransferase plays an important role in plant growth, salt/osmotic stress tolerance and seed lipid storage [35]. In this study, we found that the CoA content was significantly downregulated in the SS species under salt stress, while no significant difference in the CoA content was observed between the ST control and salt-treated groups (Additional file 1: Figure S6C). Based on these findings, we hypothesized on a possible mechanism of salt tolerance regulation in *Zygophyllum* plants, in which the overexpressed BCATs in the SS salt-treated group could transform 3-methyl-2-oxobutanoate to valine, resulting in a decreased CoA content, which reduced the plants’ salt tolerance (Fig. 9). However, whether exogenous CoA or 3-methyl-2-oxobutanoate might improve the salt tolerance of *Zygophyllum* and the molecular mechanism by which BCATs regulate salt tolerance of *Zygophyllum* needs to be investigated further in future studies.

## Conclusions

The harsh living environment of *Zygophyllum* species determines their excellent genetic adaptations to abiotic stress. In this study, we analyzed the differentially expressed genes and differentially enriched metabolites of two *Zygophyllum* species (the salt-tolerant species *Z. brachypterum* and the salt-sensitive species *Z. fabago*) after salt stress by transcriptomic and metabolomic approaches. We found that the expression of 2 BCAT genes among the 11 overlapping DEGs was significantly induced by salt. These genes were significantly enriched in valine, leucine and isoleucine biosynthesis, as well as the pantothenate and CoA biosynthesis pathways. Moreover, the overlapping differentially abundant metabolites between the ST and SS species were significantly enriched in the pantothenic acid and CoA biosynthesis pathways. Although there was no significant difference in CoA content in the ST species after salt treatment, the CoA content of the SS species decreased significantly after salt treatment. Our results indicate that 2 BCAT genes might affect CoA biosynthesis to regulate the salt tolerance of *Zygophyllum* species, possibly contributing a new pathway for improving plant response to salt stress.

## Methods

### Seed germination of *Zygophyllum* spp

Seeds of *Zygophyllum brachypterum*, *Zygophyllum obliquum* and *Zygophyllum fabago* were collected from plants in Xinjiang province, China, and the geographical information of the collection sites was recorded in Additional file 1: Table S3. As *Zygophyllum* spp. are not endangered, collection of samples for scientific purposes was permitted by local legislation. Professor Li Zhijun, botany major of Tarim University, and Professors Huang Wenjuan and Qiu Aijun, taxonomy major of Tarim University, participated in the identification of specimens. The voucher specimens (TD-00153: *Z. brachypterum*, TD-01776: *Z. obliquum*, and TD-00205: *Z. fabago*) are stored in the herbarium of Tarim University, and the data related to the specimens are included in the database of wild plant germplasm resources of the Tarim basin (internal website, not yet open to the public; data available from the corresponding author upon reasonable request).

For seed germination, vermiculite and perlite were mixed at a ratio of 3:1, after which about 100 seeds were buried in the culture substrate, and an adequate amount of water was added to allow the substrate to fully hydrate. The pots were incubated at 26 °C under a 16 h/d light regimen, and water was added at 8:00 AM and 17:00 PM every day to ensure the normal germination of the seeds.

### Histological analysis

For paraffin sectioning, main stems and leaves at the same position on the main stems of *Z. brachypterum*, *Z. obliquum* and *Z. fabago* were collected at the mature stage of the plants. Paraffin sections were made as described by Ikeda-Kawakatsu et al. [36], with minor modifications as follows: The samples were fixed with FAA (formalin/glacial acetic acid/70% ethanol (1:1:18)) at 4 °C overnight, then dehydrated by a graduated ethanol series and a xylene series, and finally embedded in Paraplast Plus and sectioned into 6-μm slices using a rotary microtome. The sections were stained with safranin O-fast green and observed under a standard optical microscope.

### Salt treatment of *Zygophyllum* seedlings

Before salt treatment, seedlings at the 10-leaf stage were fed with Hoagland nutrient solution [37] for one week to promote growth. In order to reduce the impact of NaCl, a gradual stress treatment was adopted. The indicated amounts of NaCl were added to Hoagland nutrient solution. The treatments were begun at 50 mM, and after 3 d of adaptation the second stress treatment (100 mM) was applied, until the concentration of NaCl in the Hoagland nutrient solution reached the concentration required for each final treatment. The phenotypic analysis and assessment of physiological indices were performed two weeks after treatment with 200 mM NaCl. Three replicates were performed for each treatment.

### Assessment of physiological indices

Leaves on the same part of the main stem were selected for the assessment of physiological indices. SOD activity was determined using the nitro-blue tetrazolium (NBT) method [38]. POD enzyme activity was determined using the guaiacol method [39]. The CAT content was determined using the ultraviolet absorption method according to Beers and Sizer [40]. Malondialdehyde (MDA) was determined following Dhindsa et al. [41]. The content of chlorophyll was determined using the acetone method [42]. The content of proline was determined according to Bates et al. [43].

### RNA preparation and sequencing

For RNA-seq and metabolite profiling, seedlings of *Z. brachypterum*, *Z. obliquum* and *Z. fabago* at the 10-leaf stage were treated with 150 mM NaCl for 24 h. Total RNA of leaves (on the same part of the main stem) and roots was isolated using the TRIzol reagent (Invitrogen, CA, USA), and treated with DNase I (Invitrogen) for 30 min at 37 °C. RNA degradation and contamination was monitored on 1% agarose gels. RNA integrity was assessed using the RNA Nano 6000 Assay Kit of the Agilent Bioanalyzer 2100 system (Agilent Technologies, CA, USA). A total amount of 1.5 μg RNA per sample was used to generate sequencing libraries using the NEBNext® Ultra™ RNA Library Prep Kit for Illumina® (NEB, USA). cDNA synthesis, Illumina sequencing and quality control were performed as described by Jiang et al. [44].

Transcriptome assembly was accomplished using Trinity [45] with min_kmer_cov set to 2 and all other parameters set to default. Alternative splicing, allele, different copies of the same gene, homelog, ortholog, etc. were assigned to the same gene because they had the same sequence source. Gene expression levels were estimated using the RSEM software package. The FPKM method was used to calculate the expression levels. Genes with *P*-values < 0.05 according to the DESeq R package (1.10.1) were assigned as differentially expressed. Gene Ontology (GO) enrichment analysis of the differentially expressed genes (DEGs) was implemented using Wallenius non-central hyper-geometric distribution in the GOseq R package [46]. KOBAS software [47] was used to test the statistical enrichment of differentially expressed genes in KEGG pathways.

### Quantitative real-time PCR

RNA samples for RNA-seq were reverse-transcribed into cDNA using M-MLV reverse transcriptase (Promega). The PCR reactions were performed on a 7500 qRT-PCR system (Applied Biosystems) according to the manufacturer’s instructions. The homologous genes of *Arabidopsis*, *Zygophyllum* Actin gene (Cluster-113,084.173931), Elf1 gene (Cluster-113,084.196164), and Tubulin gene (Cluster-113,084.111404) were used as internal references, and the gene expression levels were normalized to the geometric average of these internal reference genes as described in previous work [48]. Three biological replicates were performed for each sample. The primers for qRT-PCR are listed in Additional file 1: Table S4.

### Metabolite profiling

Leaves of *Z. brachypterum*, *Z. obliquum* and *Z. fabago* treated with 150 mM NaCl for 24 h and those of untreated control plants were harvested for untargeted metabolite profiling based on the LC-MS/MS platform of BIOTREE biotechnology co., LTD. Samples comprising 200 mg were was suspended in either 1.0 ml pure methanol or 1.0 ml 75% aqueous methanol for the extraction of lipidsoluble and water-soluble metabolites, respectively. After homogenization in a ball mill, the samples were ultrasonicated on ice and incubated for 1 h at − 20 °C to precipitate proteins. Then, the samples were centrifuged at 12,000×g for 15 min at 4 °C, the supernatant transferred into a fresh EP tube, and dried in a vacuum concentrator without heating. The dried powder was reconstituted in extraction liquid reconstitution, vortexed 30 s, and sonicated 10 min, centrifuged at 12,000×g for 15 min at 4 °C. Finally, the supernatant was transferred into a fresh 2 mL LC/MS glass vial for the UHPLC-QTOF-MS analysis. LC-MS/MS analyses were performed using an UHPLC system (1290, Agilent Technologies) with a UPLC BEH Amide column coupled to a TripleTOF 6600 (Q-TOF, AB Sciex). Raw MS data were processed according to Liao et al. [49]. The quantity of each compound was determined by the ratio of the peak area of a particular component to the peak area of 2-Chloro-L-phenylalanine that was used as an internal control. Additionally, differentially abundant metabolites were extracted based on the values retrieved by Student’s *t*-test (*P* < 0.05) and VIP values exceeding 1 (variable importance in the projection, VIP > 1). The Venn diagrams were drawn using the Venn tool on the bioinformatics analysis website (http://bioinformatics.psb.ugent.be/webtools/Venn/). Pathway enrichments were calculated using the MetaboAnalyst website (https://www.metaboanalyst.ca/) [50], in conjunction with the *Arabidopsis* KEGG databases.

### Statistical analysis

For multiple comparisons, one-way ANOVA with Duncan’s multiple range test (comparison of all lines) was performed using SPSS software version 23 (IBM Corp., USA). Different letters indicate significant differences at *P* < 0.05.

## Supplementary information

**Additional file 1:** **Figure S1.** Distribution of transcripts and gene sequences. **Figure S2.** Volcano maps of the DEGs. DEGs in the leaves of the ST species (**A**), in the leaves of the SS species (**B**), in roots of ST (**C**) and in roots of SS (**D**) in the control and salt-treated group. The scattered blue dots represent genes with no significant differences, red dots represent significantly up-regulated genes, and green dots represent significantly down-regulated genes. **Figure S3.** Analysis of DEGs between the ST and SS species in leaves. **A**, Expression patterns of the overlapping DEGs in leaves between ST and SS under control and salt-treatment conditions. The heatmap presents normalized FPKM expression values. **B**, Analysis of GO terms based at the overlapping DEGs. “*” indicated DEGs significantly enriched at *p* < 0.05. **C**, KEGG pathway analysis based at the overlapping DEGs. **Figure S4.** Analysis of DEGs between the ST and SS species in roots. **A**, Expression patterns of the overlapping DEGs in roots between ST and SS in the control and salt-treated group. The heatmap presents normalized FPKM expression values. **B**, Analysis of GO terms based at the overlapping DEGs. “*” indicated DEGs significantly enriched at *p* < 0.05.**C**, KEGG pathway analysis based at the overlapping DEGs. **Figure S5.** Validation of the expression of selected DEGs in leaves and roots by qRT-PCR. FPKM values of selected DEGs in leaves of the ST and SS species (**A**) and validation of the expression of these DEGs by qRT-PCR (**B**). FPKM values of selected DEGs in roots of the ST and SS species (**C**) and validation of the expression of these DEGs by qRT-PCR (**D**). **Figure S6.** Differentially abundant metabolites in the CoA pathway**.** Content of 3-methyl-2-oxobutanoate (**A**), valine (**B**) and CoA (**C**) in the control and salt-treated groups of the ST and SS species. **Figure S7.** Correlation analysis between DEGs and leaf physiological characteristics of SS species under control and salt stress conditions. A total of 134 DEGs and 42 metabolites were used for correlation matrix analysis. The coordinated shift in metabolites was evaluated by pair correlation analysis. The correlation coefficient (positive or negative) is represented by virtual color as indicated in the color key. **Table S1.** Quality assessment of sample sequencing output data. **Table S2.** Statistical analysis of transcripts and gene sequence lengths. **Table S3.** Geographical information of the collection sites of *Zygophyllum* plants used in this study. **Table S4.** Primers used in this study

## Data Availability

The RNA-Sequencing raw data have been deposited to the National Centre for Biotechnology Information (NCBI) BioProject database under accession number PRJNA560976. All the supporting data are included as additional files.

## Abbreviations

ABA: Abscisic acid
BCAT: Branched-chain-amino-acid aminotransferase
CAT: Catalase
DEG: Differentially expressed gene
MAPK: Mitogen-activated protein kinase
MDA: Malondialdehyde
NBT: Nitro-blue tetrazolium
POD: Peroxidase
qRT-PCR: Quantitative real-time PCR
SS: Salt sensitive
ST: Salt tolerance
SOS: Salt overly sensitive
SOD: Superoxide dismutase
SEM: Scanning electron microscopy
