# Supplementary material for: Transcriptomic and metabolomic analysis reveals the role of CoA in the salt tolerance of Zygophyllum spp
Source: BMC Plant Biol. 2020 Jan 6;20:9. doi: 10.1186/s12870-019-2226-8 (PMC6945424; doi:10.1186/s12870-019-2226-8)
Supplement: Supplementary file 1 — Additional file 1: Figure S1. Distribution of transcripts and gene sequences. Figure S2. Volcano maps of the DEGs. DEGs in the leaves of the ST species (A), in the leaves of the SS species (B), in roots of ST (C) and in roots of SS (D) in the control and salt-treated group. The scattered blue dots represent genes with no significant differences, red dots represent significantly up-regulated genes, and green dots represent significantly down-regulated genes. Figure S3. Analysis of DEGs between the ST and SS species in leaves. A, Expression patterns of the overlapping DEGs in leaves between ST and SS under control and salt-treatment conditions. The heatmap presents normalized FPKM expression values. B, Analysis of GO terms based at the overlapping DEGs. “*” indicated DEGs significantly enriched at p < 0.05. C, KEGG pathway analysis based at the overlapping DEGs. Figure S4. Analysis of DEGs between the ST and SS species in roots. A, Expression patterns of the overlapping DEGs in roots between ST and SS in the control and salt-treated group. The heatmap presents normalized FPKM expression values. B, Analysis of GO terms based at the overlapping DEGs. “*” indicated DEGs significantly enriched at p < 0.05.C, KEGG pathway analysis based at the overlapping DEGs. Figure S5. Validation of the expression of selected DEGs in leaves and roots by qRT-PCR. FPKM values of selected DEGs in leaves of the ST and SS species (A) and validation of the expression of these DEGs by qRT-PCR (B). FPKM values of selected DEGs in roots of the ST and SS species (C) and validation of the expression of these DEGs by qRT-PCR (D). Figure S6. Differentially abundant metabolites in the CoA pathway. Content of 3-methyl-2-oxobutanoate (A), valine (B) and CoA (C) in the control and salt-treated groups of the ST and SS species. Figure S7. Correlation analysis between DEGs and leaf physiological characteristics of SS species under control and salt stress conditions. A total of 134 DEGs and 42 metabolites w [file 12870_2019_2226_MOESM1_ESM.docx]

Additional files


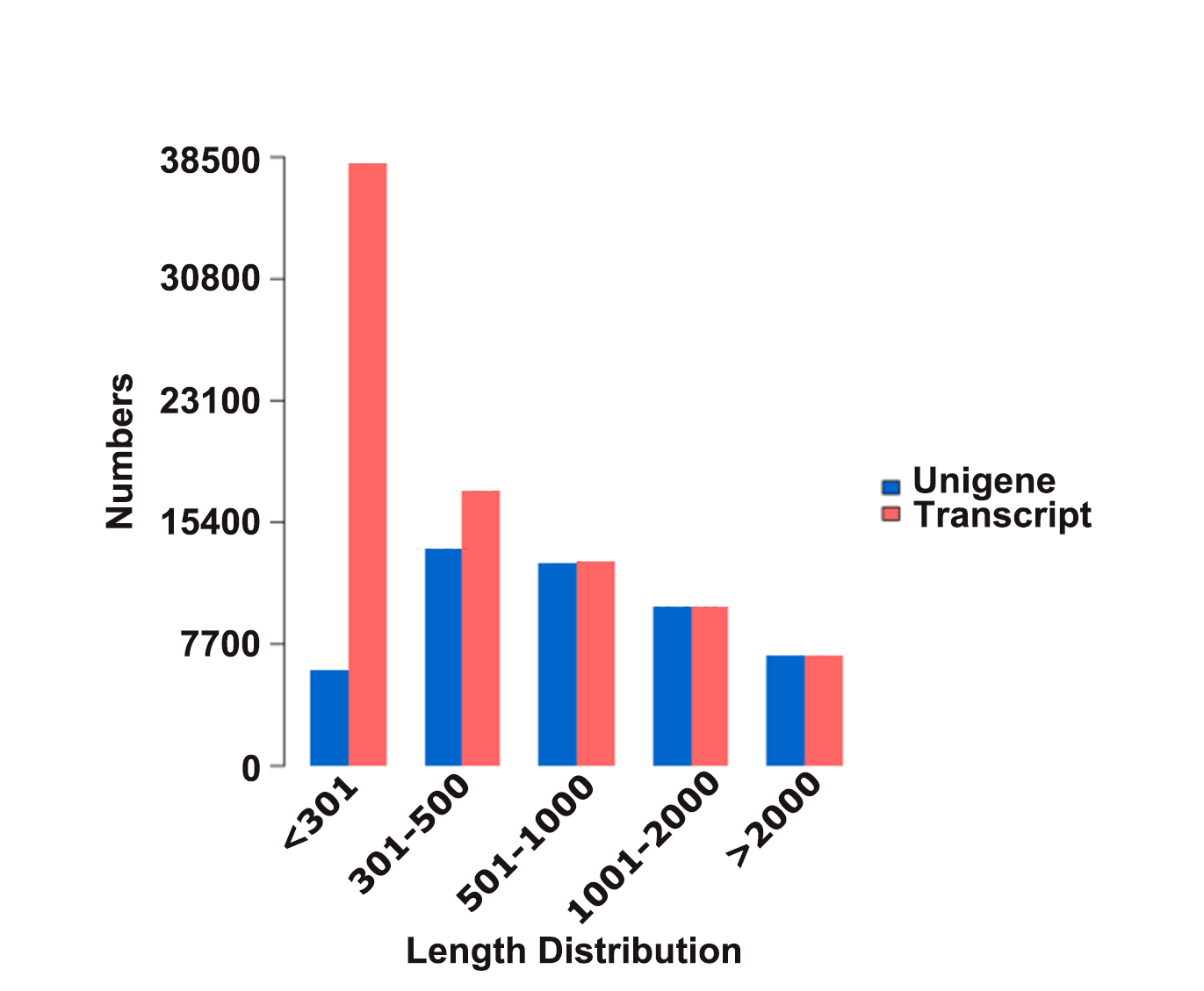


**Additional file 1: Figure S1.** **Distribution of transcripts and gene sequences.**


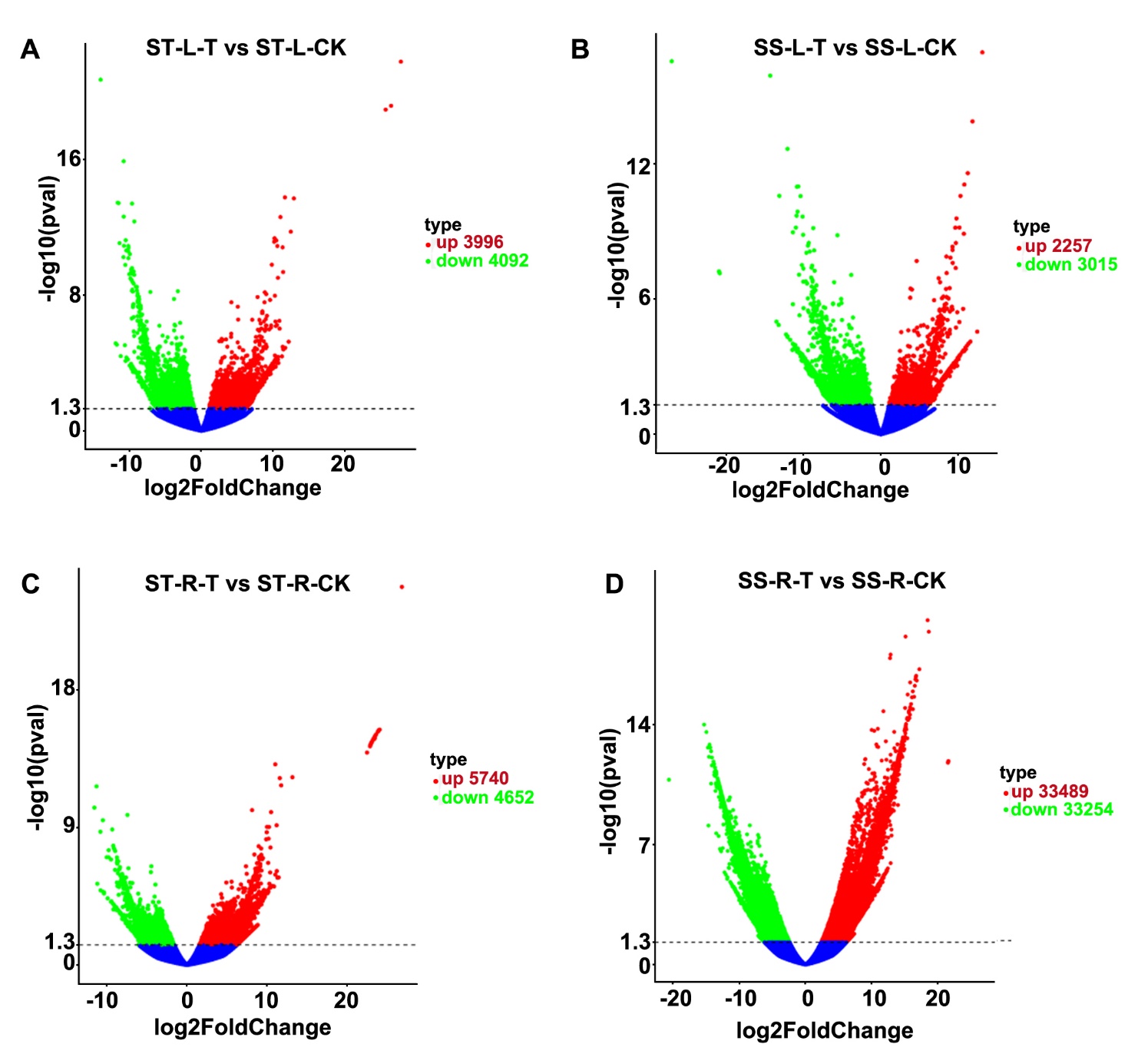
**Additional file 2: Figure S2. Volcano maps of the DEGs.** DEGs in the leaves of the ST species (**A**), in the leaves of the SS species (**B**), in roots of ST (**C**) and in roots of SS (**D**) in the control and salt-treated group. The scattered blue dots represent genes with no significant differences, red dots represent significantly up-regulated genes, and green dots represent significantly down-regulated genes.

**
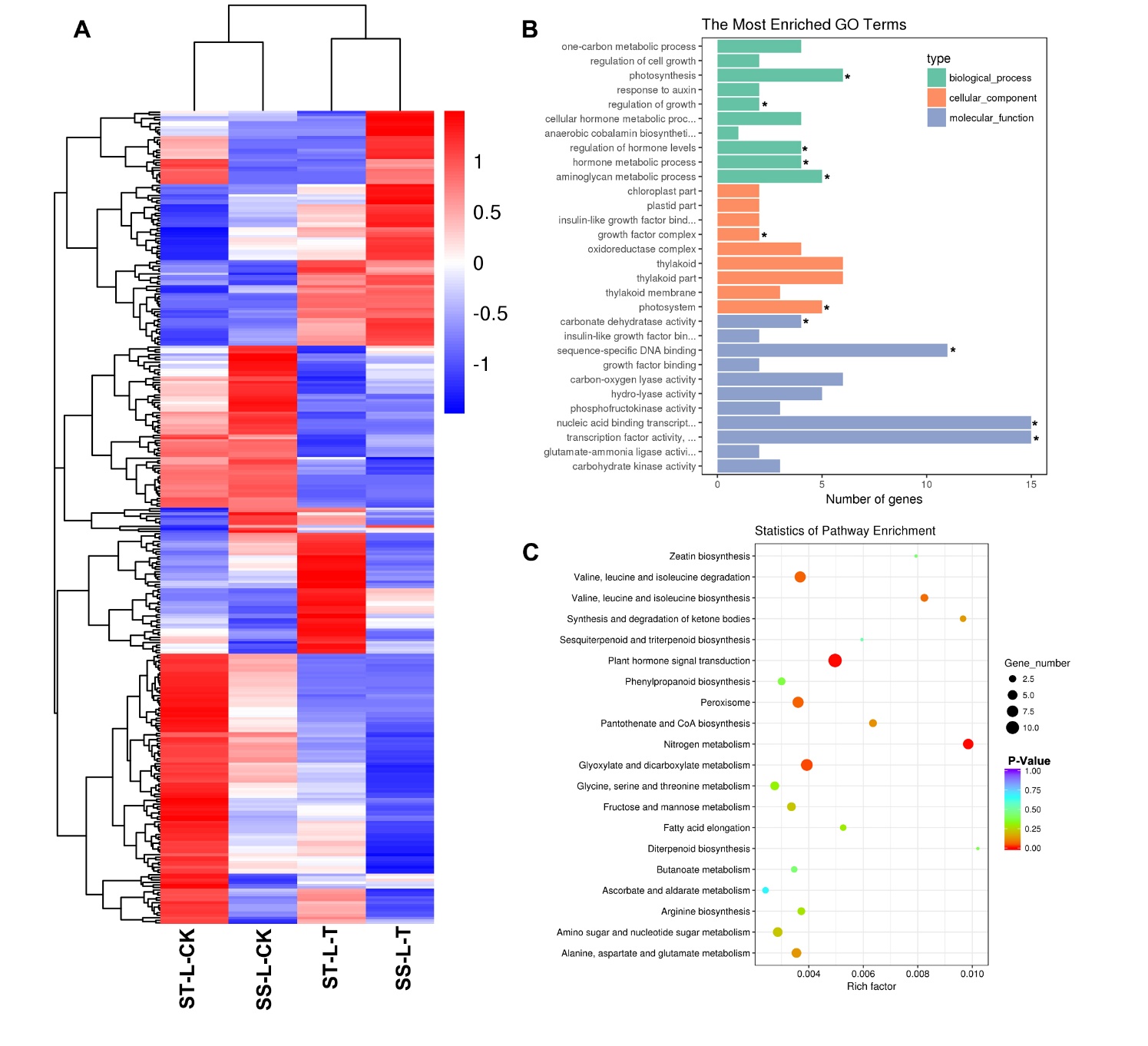
**

**Additional file 3: Figure S3. Analysis of DEGs between the ST and SS species in leaves. A**, Expression patterns of the overlapping DEGs in leaves between ST and SS under control and salt-treatment conditions. The heatmap presents normalized FPKM expression values. **B**, Analysis of GO terms based at the overlapping DEGs. “*” indicated DEGs significantly enriched at *p* < 0.05. **C**, KEGG pathway analysis based at the overlapping DEGs.


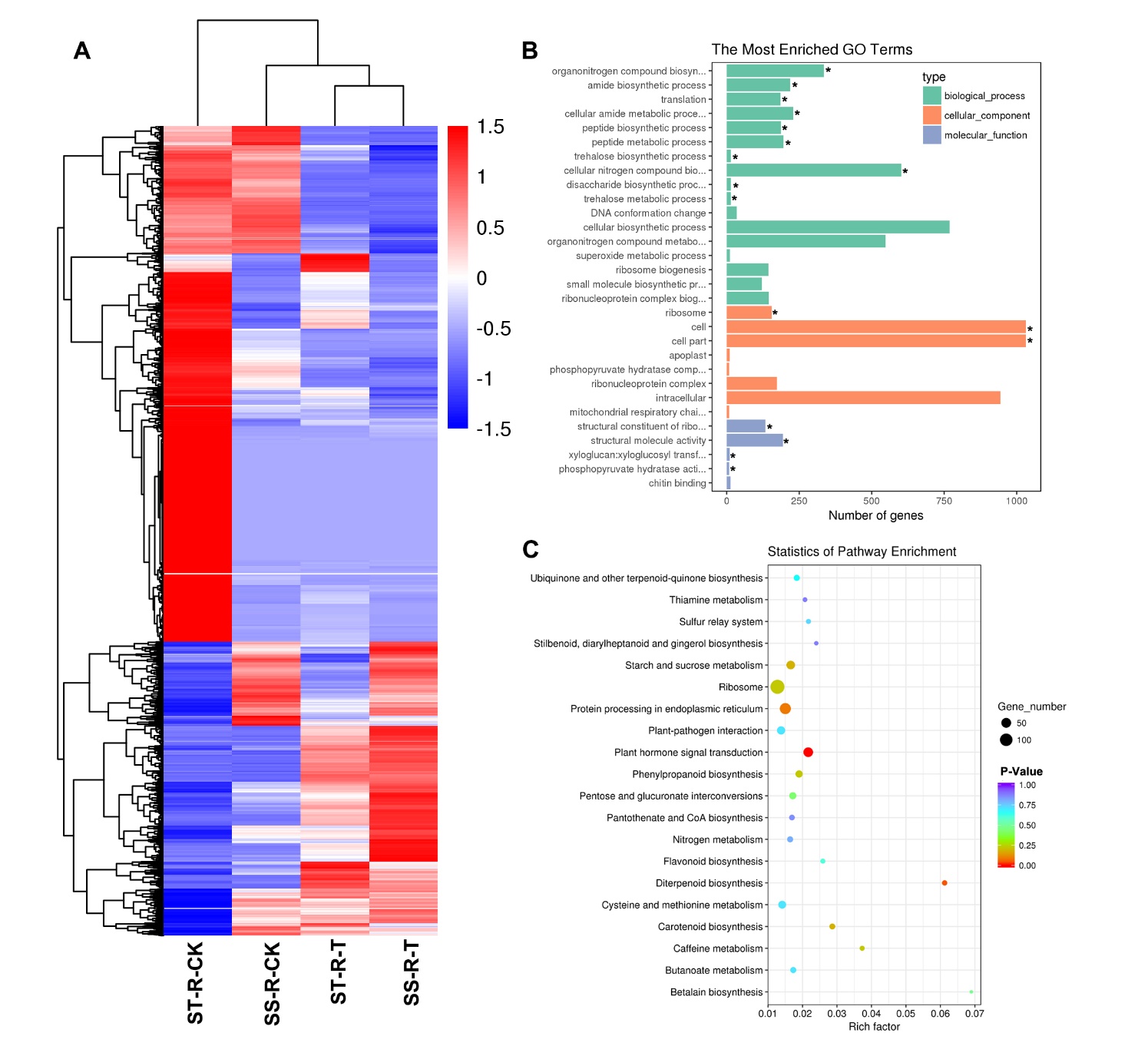


**Additional file 4: Figure S4. Analysis of DEGs between the ST and SS species in roots. A**, Expression patterns of the overlapping DEGs in roots between ST and SS in the control and salt-treated group. The heatmap presents normalized FPKM expression values. **B**, Analysis of GO terms based at the overlapping DEGs. “*” indicated DEGs significantly enriched at *p* < 0.05. **C**, KEGG pathway analysis based at the overlapping DEGs.


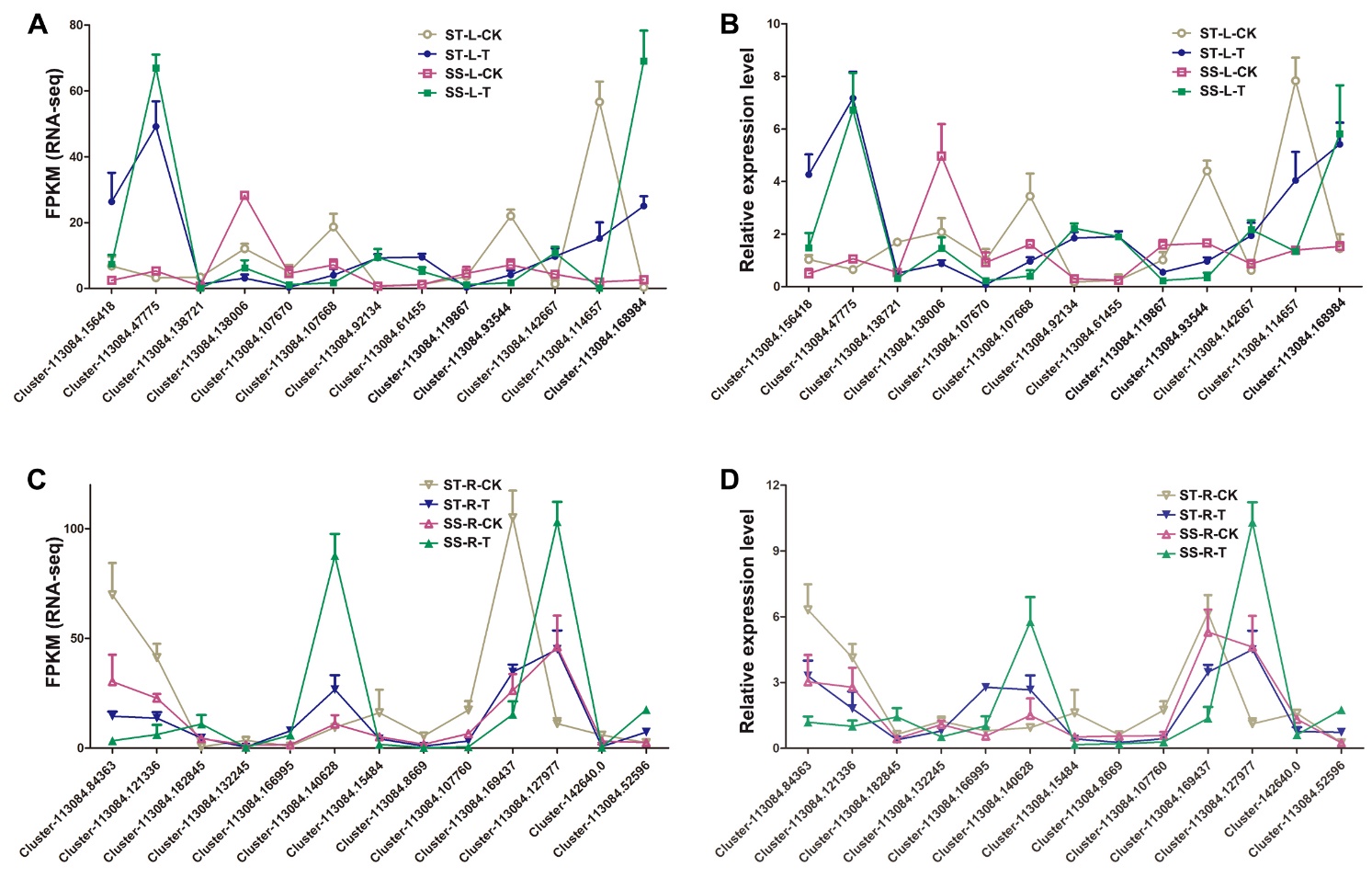


**Additional file 5: Figure S5. Validation of the expression of selected DEGs in leaves and roots by qRT-PCR.** FPKM values of selected DEGs in leaves of the ST and SS species (**A**) and validation of the expression of these DEGs by qRT-PCR (**B**). FPKM values of selected DEGs in roots of the ST and SS species (**C**) and validation of the expression of these DEGs by qRT-PCR (**D**).

**
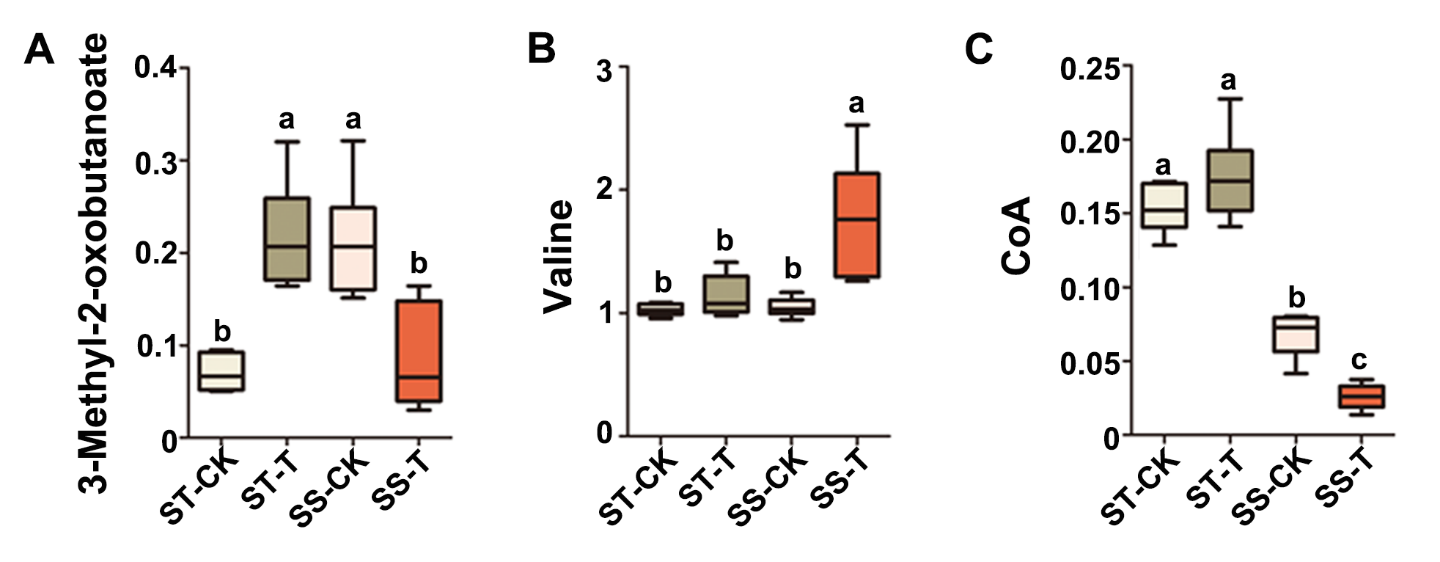
**

**Additional file 6: Figure S6. Differentially abundant metabolites in the CoA pathway.** Content of 3-methyl-2-oxobutanoate (**A**), valine (**B**) and CoA (**C**) in the control and salt-treated groups of the ST and SS species.


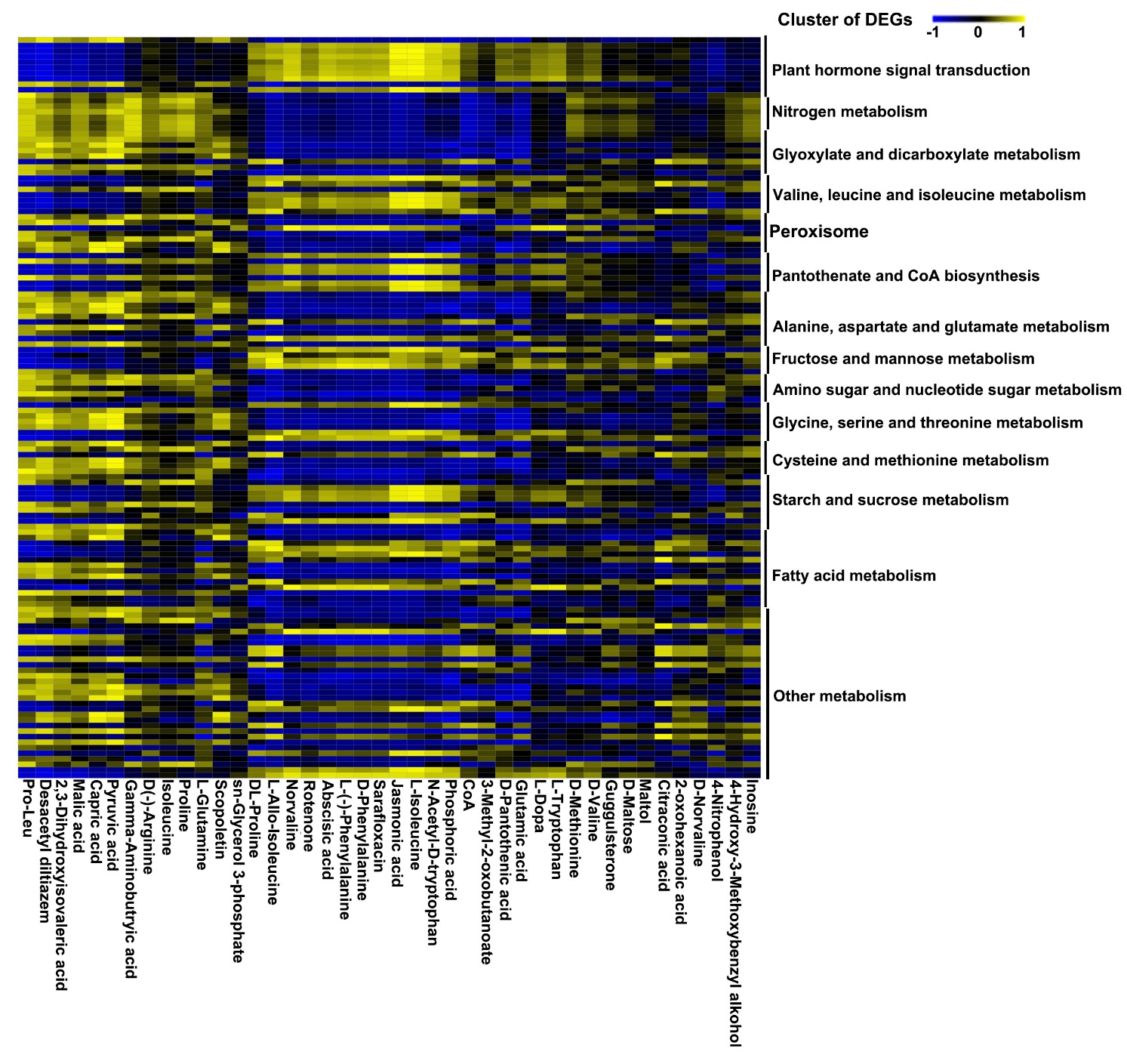


**Additional file 7: Figure S7. Correlation analysis between DEGs and leaf physiological characteristics of SS species under control and salt stress conditions.** A total of 134 DEGs and 42 metabolites were used for correlation matrix analysis. The coordinated shift in metabolites was evaluated by pair correlation analysis. The correlation coefficient (positive or negative) is represented by virtual color as indicated in the color key.

**Additional file 8: Table S1. Quality assessment of sample sequencing output data.**

| **Sample** | **Raw Reads** | **Clean Reads** | **Clean Bases** | **Error (%)** | **Q20 (%)** | **Q30 (%)** | **GC Content (%)** | **Total mapped** |
| --- | --- | --- | --- | --- | --- | --- | --- | --- |
| ST_R_CK1 | 46478876 | 44730670 | 6.71G | 0.03 | 96.23 | 90.69 | 42.69 | 35855164(80.16%) |
| ST_R_CK2 | 52334182 | 51066084 | 7.66G | 0.03 | 96.61 | 91.45 | 42.27 | 41349240(80.97%) |
| ST_R_CK3 | 51264850 | 49935194 | 7.49G | 0.03 | 96.53 | 91.31 | 42.43 | 39635094(79.37%) |
| ST_R_T1 | 58632962 | 57304240 | 8.6G | 0.03 | 96.2 | 90.83 | 42.36 | 43427750(75.78%) |
| ST_R_T2 | 52590978 | 50784058 | 7.62G | 0.03 | 96.07 | 90.65 | 42.11 | 38975134(76.75%) |
| ST_R_T3 | 54732434 | 52966474 | 7.94G | 0.03 | 96.41 | 91.1 | 42.97 | 42820330(80.84%) |
| ST_L_CK1 | 56931754 | 56125772 | 8.42G | 0.03 | 96.69 | 91.55 | 43.69 | 46996284(83.73%) |
| ST_L_CK2 | 55644456 | 54493598 | 8.17G | 0.03 | 96.82 | 91.86 | 42.89 | 45078886(82.72%) |
| ST_L_CK3 | 59291686 | 57855870 | 8.68G | 0.03 | 96.55 | 91.33 | 42.99 | 47109922(81.43%) |
| ST_L_T1 | 48340892 | 46421578 | 6.96G | 0.03 | 96.54 | 91.3 | 41.91 | 37852678(81.54%) |
| ST_L_T2 | 56063422 | 54313360 | 8.15G | 0.03 | 96.48 | 91.17 | 42.78 | 44110736(81.22%) |
| ST_L_T3 | 43801614 | 42771698 | 6.42G | 0.03 | 96.4 | 91.02 | 42.52 | 33566722(78.48%) |
| SS_R_CK1 | 62539186 | 60862128 | 9.13G | 0.03 | 96.37 | 90.96 | 42.59 | 47125370(77.43%) |
| SS_R_CK2 | 49774820 | 48612728 | 7.29G | 0.03 | 96.6 | 91.39 | 43.28 | 38729254(79.67%) |
| SS_R_CK3 | 51683168 | 50146632 | 7.52G | 0.03 | 96.68 | 91.53 | 43.02 | 39703434(79.17%) |
| SS_R_T1 | 45684936 | 44601234 | 6.69G | 0.03 | 96.36 | 91.04 | 42.8 | 34056026(76.36%) |
| SS_R_T2 | 56566776 | 54837670 | 8.23G | 0.03 | 94.96 | 88.86 | 40.95 | 34343822(62.63%) |
| SS_R_T3 | 46429322 | 44906686 | 6.74G | 0.03 | 96.6 | 91.43 | 42.95 | 34823798(77.55%) |
| SS_L_CK1 | 48093226 | 46791602 | 7.02G | 0.03 | 96.45 | 91.06 | 43.04 | 37647494(80.46%) |
| SS_L_CK2 | 52963042 | 51761602 | 7.76G | 0.03 | 96.69 | 91.56 | 42.73 | 40322842(77.90%) |
| SS_L_CK3 | 46329796 | 44955556 | 6.74G | 0.03 | 96.65 | 91.45 | 43.14 | 35462218(78.88%) |
| SS_L_T1 | 65198904 | 63478036 | 9.52G | 0.03 | 96.57 | 91.24 | 42.9 | 50940890(80.25%) |
| SS_L_T2 | 48560152 | 47083324 | 7.06G | 0.03 | 96.64 | 91.48 | 42.57 | 37220552(79.05%) |
| SS_L_T3 | 57377076 | 55770126 | 8.37G | 0.03 | 96.43 | 91.02 | 43.02 | 44693160(80.14%) |

**Additional file 9: Table S2. Statistical analysis of transcripts and gene sequence lengths.**

|  | **Min Length** | **Mean Length** | **Median Length** | **Max Length** | **N50** | **N90** | **Total Nucleotides** |
| --- | --- | --- | --- | --- | --- | --- | --- |
| Transcripts | 201 | 709 | 335 | 37056 | 1354 | 256 | 607422945 |
| Genes | 201 | 1038 | 634 | 37056 | 1664 | 430 | 517330868 |

**Additional file 10: Table S3. Geographical information of the collection sites of *Zygophyllum* plants used in this study.**

| **Specific name** | **Collection place** | **Longitude (E)** | **Latitude (N)** | **Altitude (m)** |
| --- | --- | --- | --- | --- |
| *Zygophyllum brachypterum* Kanitz | Tiereke town, aksu baicheng county, xinjiang province, China | 81° 36′ 31.5″ | 41° 52′ 2.6″ | 1522.7 |
| *Zygophyllum obliquum* Popov | Aheqi county, xinjiang province, China | 78° 23′ 29.7″ | 40° 54′ 40.6″ | 2096.7 |
| *Zygophyllum fabago* Linn | Kuqa County, xinjiang province, China | 83° 02′ 55.0″ | 41° 58′ 41.4″ | 1351.8 |

**Additional file 11: Table S4. Primers used in this study.**

| **Primers** | **Forward sequence (5' - 3')** | **Reverse sequence (5' - 3')** |
| --- | --- | --- |
| *Elf1* | AGATGATTCCGACCAAACCCA | GGAACACGAAACAGAAGGCAG |
| *Actin* | TAGATGGCTGGAACAGAACTT | GTTGCTATTGATTATGAGAAGG |
| *Tublin* | CAGTAGGTGGCTGGTAGTTG | TCATACGCTCCTGTCATCTCT |
| q113084.50397 | GGCTGTTGCGAGAGATGCTTA | CCCCACTGTAGAGACTCCTTTCC |
| q113084.77458 | GTTGTATTCCACGCTACTGTC | TAATTCTAACCTCACCCCCTC |
| q113084.52599 | TGCTTCTGGCTTGGACGACTA | GCCGACGGGCTAATCTCAATA |
| q113084.128891 | GCAGAGGAAGCATCAAGACAA | TCAGGTGAGTAGCCAAAGGAA |
| q113084.61260 | TGTGTGTCAAGCACCCGTTTT | CAACATCAACAAAGAGGTCGCC |
| q113084.125321 | ATTTTACTGATGTTGGGGGTG | CGTTGGCTTTCTGATTTTCG |
| q113084.129563 | GAAGAAGTTGTAAACCGTGAG | TTTGTATGGGCTGGAGAAT |
| q113084.93940 | GGGATGTGCTTATTCTGGCTA | CTCTAAAATGGGGTCTACTTGTTC |
| q113084.52595 | GTATGACCGTGCTGCTCGTAG | CTGGTGATTCCTTCAAGTATGG |
| q113084.22649 | TTTCCTGCCCTTTTTCTTCT | ATTCTCACTCCTCTTTTTGCTAA |
| q113084.97367 | AGGCAGTTTGGTTCATTGGAC | TGCTATGACCTCCCATTGTTG |
| q113084.138006 | AATGACAGCAAAAGTCCCAAC | TGAGTTTGAGAGGATGGTTGA |
| q113084.107670 | AACCCCATTTATCATCCCTAC | CAGTCACTCTCTACCACATCG |
| q113084.107668 | ATGTGGTAGAGAGCGACTGGG | TATGCGAAAAAAACGAAAGGG |
| q113084.92134 | GATGACAAGGGGAGAAGATAC | GGTAAAGAACTGTGGAATGGT |
| q113084.61455 | CCTCTAATGTAAGATGGGCGG | TGAAGCGTGTAAAAAGGCAAC |
| q113084.119867 | GCATAAGAAAGGACTCGCATA | ATGAAGCAAGTGGCATCTAAT |
| q113084.93544 | GATAGGTCTAAAAAGAGGAGG | ATGGTTCTACGCTTCTACTGC |
| q113084.114657 | ACAACAGCCATCACAGCCTCC | ATCGGATGTCCAGTAGAAGGG |
| q113084.168984 | ATACGCAGGATAATAAGGTGA | ATCCTTTTCATCCAACTCAGA |
| q113084.84363 | ATGAAAGCAACAGCAGCAAAA | ACGAATACCTCCAATCCACAA |
| q113084.121336 | ATAAAATGCCAACCATCTCTAC | CAATGTCATCATCACCCAGG |
| q113084.182845 | ATGGAACATCCCCAACAATCA | CTGGGCTGAGGCAGATAAAGT |
| q113084.132245 | TGAGATTTGAGGGAGAAGATT | TGAAACTCTATCAGGTCTTGC |
| q113084.166995 | GACTCTCTTTCCTCTTCTCCG | AGATACAGAATCATTGTTGCC |
| q113084.140628 | ATTTCAGGTGGATTTATGTGC | TTGTCTTCCCTTCTGTAACTC |
| q113084.15484 | GCCTACAATGAACTTGAACCA | ACGAAGACCAATGTGATAATG |
| q113084.8669 | CATAGACAAAAACTTGAGCCA | ATGGATGGTTACATACAGGTG |
| q113084.107760 | CCTTCAACCATCCCTATCTTT | ACTGCTGAGGAGGTGACATTG |
| q113084.169437 | TCTTCACAACAACTTGATGGT | TACACTATGCCTCCTGATGC |
| q113084.127977 | GTTCTCCGAGGCTATTCCGT | ATCGTCGTCACATCATAAGTTT |
| q142640.0 | CACCCGTTTATGATGCCGTTA | ATTGAAACCCATTCGCCATCT |
| q113084.52596 | TATCAAAACAAACAATAAGCA | TTCAATCTATCACCACAACC |
